# Lateral lamina V projection neuron axon collaterals connect sensory processing across the dorsal horn of the mouse spinal cord

**DOI:** 10.1038/s41598-024-73620-4

**Published:** 2024-11-01

**Authors:** Tyler J. Browne, Kelly M. Smith, Mark A. Gradwell, Christopher V. Dayas, Robert J. Callister, David I. Hughes, Brett A. Graham

**Affiliations:** 1 School of Biomedical Sciences and Pharmacy, Faculty of Health, University of Newcastle, Callaghan, NSW 2308 Australia; 2 Hunter Medical Research Institute (HMRI), New Lambton Heights, NSW Australia; 3 Department of Neurobiology and the Pittsburgh Center for Pain Research, University of Pittsburgh, Pittsburgh, PA 15213 USA; 4 Department of Cell Biology and Neuroscience, Rutgers, The State University of New Jersey, Piscataway, NJ USA; 5 W.M. Keck Center for Collaborative Neuroscience, Rutgers, The State University of New Jersey, Piscataway, NJ USA; 6 Institute of Neuroscience Psychology, College of Medical, Veterinary and Life Sciences, University of Glasgow, Glasgow, UK

**Keywords:** Sensory processing, Optogenetics, Neuroscience, Neurophysiology, Central nervous system

## Abstract

Spinal projection neurons (PNs) are defined by long axons that travel from their origin in the spinal cord to the brain where they relay sensory information from the body. The existence and function of a substantial axon collateral network, also arising from PNs and remaining within the spinal cord, is less well appreciated. Here we use a retrograde viral transduction strategy to characterise a novel subpopulation of deep dorsal horn spinoparabrachial neurons. Brainbow assisted analysis confirmed that virally labelled PN cell bodies formed a discrete cell column in the lateral part of Lamina V (LV^lat^) and the adjoining white matter. These PNs exhibited large dendritic territories biased to regions lateral and ventral to the cell body column and extending considerable rostrocaudal distances. Optogenetic activation of LV^Lat^ PNs confirmed this population mediates widespread signalling within spinal cord circuits, including activation in the superficial dorsal horn. This signalling was also demonstrated with patch clamp recordings during LV^Lat^ PN photostimulation, with a range of direct and indirect connections identified and evidence of a postsynaptic population of inhibitory interneurons. Together, these findings confirm a substantial role for PNs in local spinal sensory processing, as well as relay of sensory signals to the brain.

**Supplementary Information:**

The online version contains supplementary material available at 10.1038/s41598-024-73620-4.

## Introduction

The spinal dorsal horn (DH) is the first central nervous system site for processing of sensory information from the body including touch, temperature, pain, and itch^1,2^. Since publication of the Gate Control Theory^3^ sensory physiologists studying nociception and pain have assembled a model where afferent neurons relay sensory input to the DH, before interneurons in the DH play a key role in shaping the level of local excitation and also receive descending input from higher brain regions that contributes to this process. Finally, specialised output projection neurons (PNs) relay processed sensory signals to supraspinal areas and elicit the sensory/affective experiences we associate with pain. While there have been many updates to this basic spinal nociceptive processing model, incorporating the nature and complexity of primary afferent input^4^ and considerable neuronal diversity within the substantia gelatinosa^5^, the role of PNs has remained largely unchanged. This overlooks the very prominent local branching (collateralisation**)** of PN axons in the spinal cord, suggesting an important but as yet ill-defined role in spinal sensory processing^6^.

Although the function of spinal PN axon collaterals has not been resolved, these structures have been noted for decades, across species, and with a variety of termination sites^6–15^. The most detailed description is in rat, showing axon collaterals densely innervate the ipsilateral DH, spanning inter- and intra-segmentally^14^ and even reach into the neck of the contralateral DH^13^. Clues on the functional significance of spinal PN axon collaterals come from collateral signalling in other CNS regions, commonly engaging inhibitory networks to provide feedback; or providing feedforward excitation to recruit other PNs and distinct circuits, priming appropriate responses or initiating plasticity and long-term changes^16–24^.

Spinal PN axon collateral function is also complicated by increasing evidence that PNs are a diverse group of spatially and functionally distinct subtypes^12,25–27^. For example, PNs can be differentiated by location into superficial and deep populations^28,29^. Experimental accessibility, advanced transgenic and viral technologies, and the nature of the superficial PNs as largely nociceptive specific has led many studies to focus on this population^25,26,30–36^. Likewise, the anatomical evidence for spinal PN axon collateralization comes almost exclusively from the superficial population. By contrast, our understanding of PNs in the deeper laminae is less advanced^37^, likely related to the difficulty recording in highly myelinated regions with patch clamp electrophysiology. Notwithstanding these challenges, we recently reported on the electrical properties of deep DH PNs and show these cells also give rise to collateral branches from the axon^12^. The current study elaborates on these insights, capitalizing on the surprising observation that injection of rAAV2 virus in the parabrachial nucleus of mice transduces a specific population of deep DH PNs that exhibit local axon collaterals. We use Brainbow multiflurophore labelling to characterise the morphology of these cells and a channelrhodopsin-2 (ChR2) assisted circuit mapping approach to study spinal collateral signalling from this population. Our results provide the first detailed description of a distinct spinoparabrachial projecting population in the lateral lamina V region, and evidence to reveal a novel axon collateral mediated pathway for linking output signals from this deep lateral PN population with circuits in the superficial DH.

## Results

This study exploited multiple viral strategies to target spinoparabrachial projection neurons, defining a distinct population in the lateral region of LV. First, the propensity of PBN retro-AAV2 serotype injection to selectively transduce lateral LV SPBNs, but not the well described larger population of lamina I SPBNs was characterised. This unexpected result was directly compared with the labelling achieved by AAV9-RFP^12^. This identified limited overlap between retro-AAV2 and AAV9 labelled SPBNs, with those cells that did exhibit co-expression concentrated in the lateral LV region (LV^lat^). The intersection of retrograde PBN rAAV2-Cre and spinal AAV-Brainbow expression allowed morphological characterisation of LV^lat^ SPBNs in the lumbar enlargement, with most exhibiting multipolar morphology, dendritic territories biased to the lateral and ventral spinal cord in the transverse plane, and extending over substantial rostrocaudal distances. Targeted recordings from ChR2-positive LV^lat^ SPBNs in spinal cord slices confirmed optogenetic control, with reliable spiking achieved during photostimulation. In vivo spinal photostimulation under anaesthesia showed that the ChR2-expressing SPBNs and axon collaterals increased the expression pERK, a neuronal activity marker, in a range of local spinal circuits. Finally, recordings from randomly selected DH neurons in acute spinal slices showed that LV^lat^ SPBNs made functional connections via local axon collaterals in 16% of recordings.

### Retro-AAV2 serotype produces brainstem labelling consistent with retrograde transduction

**Fig. 1:**
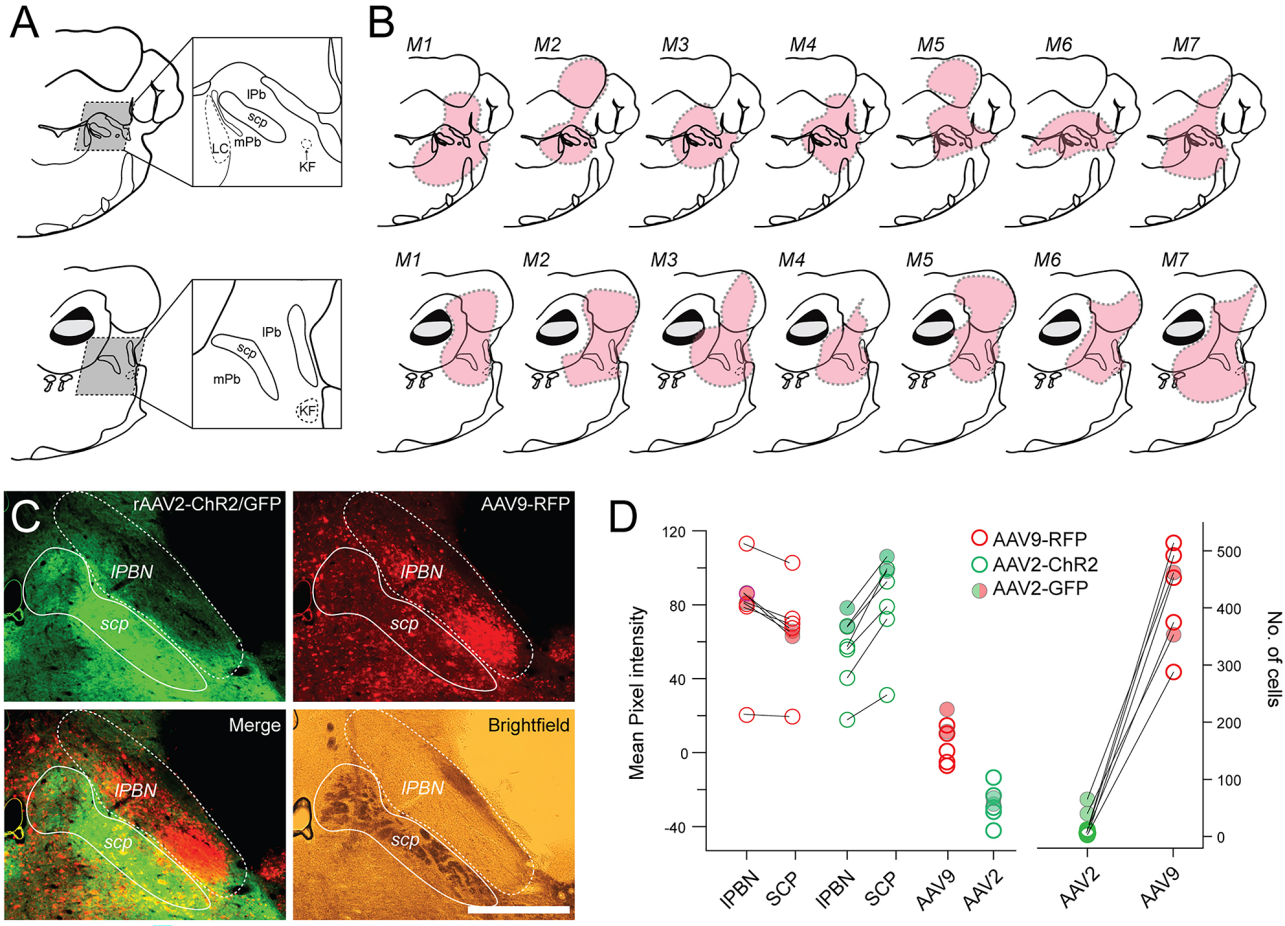
AAV serotype specific labelling of brainstem injection sites. (**A**) Left schematics show maps of two adjoining brainstem sections through the level of the parabrachial nucleus, constructed from Allen Brain Atlas^86^ in Adobe Illustrator (2024; version 28.0; https://www.adobe.com/au/products/illustrator.html). Major landmarks are annotated including the superior cerebellar peduncle (SCP) separating the medial and lateral parabrachial nuclei (mPb and lPb, respectively), the Kolliker-Fuse Nuclei (KF), and Locus Coeruleus (LC). (**B**) Brainstem maps (aligned to sections in **A**), with injection site reconstructions for 7 animals (denoted M1-M7) based on AAV9-RFP fluorescent signal (red). (**C**) Representative images show distinct labelling patterns for rAAV2-GFP (upper left) and AAV9-RFP (upper right) serotypes in the PBN. Merged image (lower left) highlight dense rAAV2 labelling of terminals across the injection site but few neurons, whereas AAV9-RFP labels many neuronal profiles across the PBN. Brightfield image (lower right) provides orientation of PBN with the prominent SCP. (**D**) Group data plots compare overall fluorescence (mean pixel intensity measured) and neuronal distributions (manually counted) from rAAV2-GFP and AAV9-RFP reflecting images in (**B**). Note more labelling in the lateral PBN compared to the superior cerebellar peduncle (SCP) for AAV9, whereas the signal is strong in the SCP compared to lPBN the for rAAV2. Strong neuronal labelling was produced by AAV9 in the lPBN, whereas AAV2-GFP and AAV2-ChR2 constructs produced negligible neuronal labelling at the injection site. Scale in A: 200 μm.

To assess how reliably the AAV2-retro serotype transduced retrograde specific expression, the number of neurons labelled by the AAV2-retro versus AAV9 was compared at the injection site (lateral PBN - lPBN, Fig. 1A,B). Consistent with a retrograde specific action for AAV2-retro but not AAV9, this comparison confirmed that few lPBN neurons were labelled with AAV2-ChR2 or AAV2-GFP constructs (Fig. 1C,D: *n* = 5 animals, 7.2 ± 4 neurons; *n* = 2 animals, 52.5 ± 18 neurons, respectively), whereas many PBN neurons were labelled with the AAV9 construct (*n* = 7 animals, 419.6 ± 82 neurons, *P* = 0.000, *N* = 14). Also consistent with enhanced retrograde tropism, AAV2-retro produced far less fluorescence in terminals within lPBN, measured by mean pixel intensity (mpi), when compared to that in the superior cerebellar peduncle (SCP) (lPBN: 55.2 ± 20.4 vs. SCP: 82.7 ± 26 mpi, difference: -27.5 ± 9 mpi, respectively). The opposite was true for the AAV9, with most injection sites showing a similar or less terminal fluorescence in SCP compared to lPBN (*n* = 7; lPBN: 75.5 ± 28 vs. SCP: 68.5 ± 26 mpi, difference: 6.9 ± 11 mpi). The difference between lPBN and SCP fluorescence reinforced this distinction in viral labelling patterns (Average lPBN vs. SCP difference: AAV9 = 6.9 ± 11mpi vs. rAAV2 = -27.5 ± 9mpi; z = 6.958, *P* = 0.00, *N* = 14). Importantly, the Rostroventolateral Medulla was not labelled in retro-AAV2, a well characterised source of descending input to the spinal cord; whereas AAV2-retro mediated cellular labelling was common in the contralateral Locus Coeruleus, ipsilateral vestibular nuclei, and paraolivary nuclei, all sources of PBN input and consistent with a retrograde action for this serotype.

### Distinct spinal distribution of rAAV2-labelled SPBNs

**Fig. 2:**
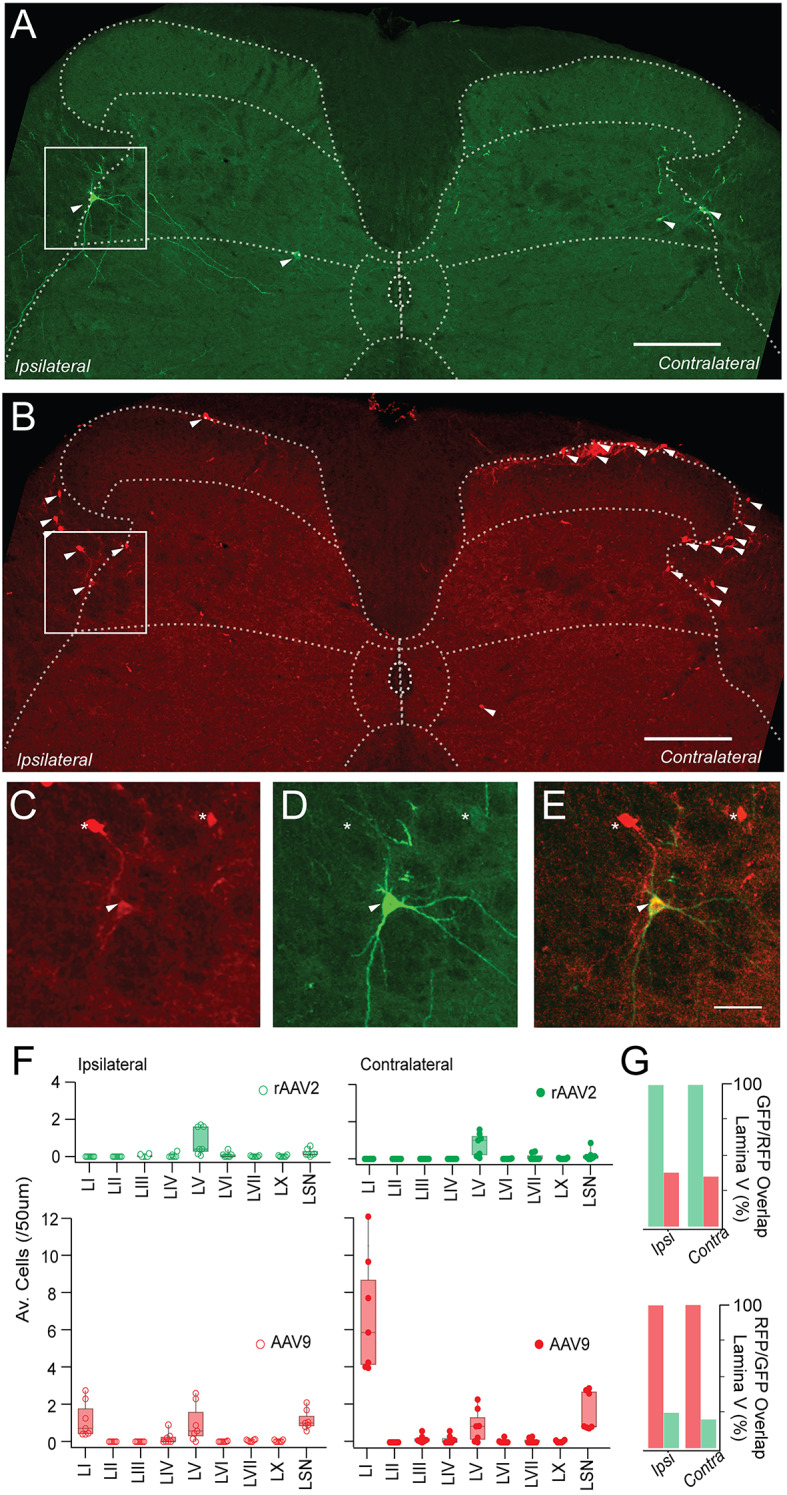
rAAV2-ChR2 identifies a discrete population of LV^lat^ SPBNs. (**A**,**B**) show a representative transverse lumbar spinal section with virally labelled SPBNs following unilateral PBN co-injection of rAAV2-ChR2/GFP and AAV9-RFP. SPBNs are indicated by arrow heads, ipsilateral and contralateral sides are relative to the PBN injection site. (**A**) rAAV2-ChR2 labels SPBNs bilaterally in the lumbar spinal cord, on the border between Lamina V and lateral spinal nucleus. (**B**) AAV9-RFP labels SPBNs with the highest density located within the contralateral lamina I region and others within lamina V, and LSN. SPBNs are also located in lamina I, LSN and lamina V of the ipsilateral spinal cord, though at lower density. (**C**–**E**) higher magnification images of AAV9-RFP, rAAV2-ChR2, and AAV9-RFP/ rAAV2-ChR2 channels overlaid, from images in (**A**,**B**) (white square). Some SPBNs are co-labelled with both viruses (arrowheads), while others were only transduced by a single virus (asterisks). (**F**) plots summarise and compare group data on SPBN numbers identified in 7 animals with dual rAAV2-GFP and AAV9-RFP PBN injections. Note the restricted rAAV2 labelling of SPBNs in lateral lamina V of the ipsilateral and contralateral DH (upper green). In contrast, AAV9 labelled a larger and much more diverse population with the greatest number of SPBNs concentrated in lamina I (lower red). (**G**) bar graphs compare rAAV2-GFP and AAV9-RFP colocalization in lamina V. Upper graph presents percentage of AAV9-RFP co-labelling in the total AAV2-ChR2 population, and lower graph shows the percentage of AAV2-ChR2 co-labelling in total AAV9-RFP population. Scale bars A & B = 100 μm, C-E = 20 μm.

Within the spinal cord, the rAAV2-ChR2/-GFP constructs labelled surprisingly few SPBNs in lumbosacral sections (*n* = 117, in 5 animals, Fig. 2A). Given this low overall number but similar overlapping distributions and densities of rAAV2-ChR2/-GFP labelled SPBNs across animals, the sample was pooled for analysis. Despite unilateral injections, rAAV2-labelled SPBN numbers were comparable between the ipsilateral (*n* = 65) and contralateral (*n* = 52) spinal cords, with the highest density observed in lamina V of the contralateral spinal cord (*n* = 30, 0.52 ± 0.50 neurons/50um), and the majority of these located in the lateral aspect of the DH, concentrated at the lateral grey/white matter border. The remaining contralateral SPBNs were infrequently distributed in other locations (LVI/VII: *n* = 16, 0.16 ± 0.21 neurons/50um; LSN: *n* = 5, 0.071 ± 0.08 neurons/50um, LVI, LX: *n* = 1, 0.01± 0.02 neurons/50um). rAAV2-labelled SPBN distributions in the ipsilateral cord were very similar to the contralateral side with the main density in lamina V (*n* = 32, 0.52 ± 0.62 neurons/50um), followed by LVI/LVII (*n* = 12, 0.13 ± 0.16 neurons/50um), and infrequent neurons within LSN, and LIII/LIV, (LSN: *n* = 8, 0.09 ± 0.06 neurons/50um; LIII-LIV: *n* = 11, 0.11 ± 0.13 neurons/50um). Finally, a single lamina I SPBN was identified in the ipsilateral spinal cord of a rAAV2-GFP animal and was the only instance across all sections analysed (*n* = 1, 0.01 ± 0.02 neuron/50um, from 91 sections).

The above rAAV2 SPBN labelling was directly compared with AAV9-RFP labelling as both viruses were included in PBN injections. Significantly more SPBNs were transduced by AAV9-RFP, with 1306 RFP profiles identified from the lumbosacral enlargement across 7 animals (Fig. 2B), greater than ten times those labelled by rAAV2-ChR2/-GFP. The distribution of AAV-RFP SPBNs was similar to our previous reports^12^, but starkly contrasted the rAAV2-ChR2/-GFP distribution. Most AAV9-RFP labelled cells were located in the contralateral spinal cord (*n* = 872, 66.8%) and concentrated in lamina I (*n* = 643, 6.8 ± 3.13 neurons/50um), followed by populations in the Lateral Spinal Nucleus (*n* = 130, 1.7 ± 1.05 neurons/50um), lamina V (*n* = 60, 0.87 ± 0.89 neurons/50um), and some across LIII, LIV, LVI, LVII and LX (*n* = 39; LIII: 0.17 ± 0.2 neurons/50um, LIV: 0.15 ± 0.2 neurons/50um, LVII: 0.08 ± 0.1 neurons/50um, LVI: 0.05 ± 0.1 neurons/50um: and LX: 0.04 ± 0.05 neurons/50um). A total of 289 SPBNs were identified in the ipsilateral spinal cord, with the main densities in lamina I (*n* = 84, 1.2 ± 1.0 neurons/50um), LSN (*n* = 102, 1.2 ± 0.5 neurons/50um), lamina V (*n* = 72, 0.99 ± 1.0 neurons/50um), and lamina IV (*n* = 23, 0.2 ± 0.3 neurons/50um), with negligible labelling elsewhere. Overlap between rAAV2 and AAV9 labelling was only assessed in LV^Lat^ as this was the only region to exhibit robust rAAV2-labelling (Fig. 2C–G). On the ipsilateral side, 38% (*n* = 22/61) of rAAV2-GFP SPBNs coexpressed AAV9-RFP, and conversely 24.7% (*n* = 22/89) of AAV9-RFP profiles exhibited coexpression of rAAV2-GFP. Overlapping expression was similar in the contralateral spinal cord, where 35.3% (*n* = 12/44) of rAAV2-GFP labelled SPBNs coexpressed AAV9-RFP, and 20% (*n* = 12/60) of AAV9-RFP labelled SPBNs coexpressing rAAV2-GFP. Thus, PBN injection of AAV9-RFP and rAAV2-GFP/ChR2 virus selectively identify distinct overlapping populations we collectively term LV^Lat^ SPBNs.

### LVlat SPBN incidence and morphology

**Fig. 3:**
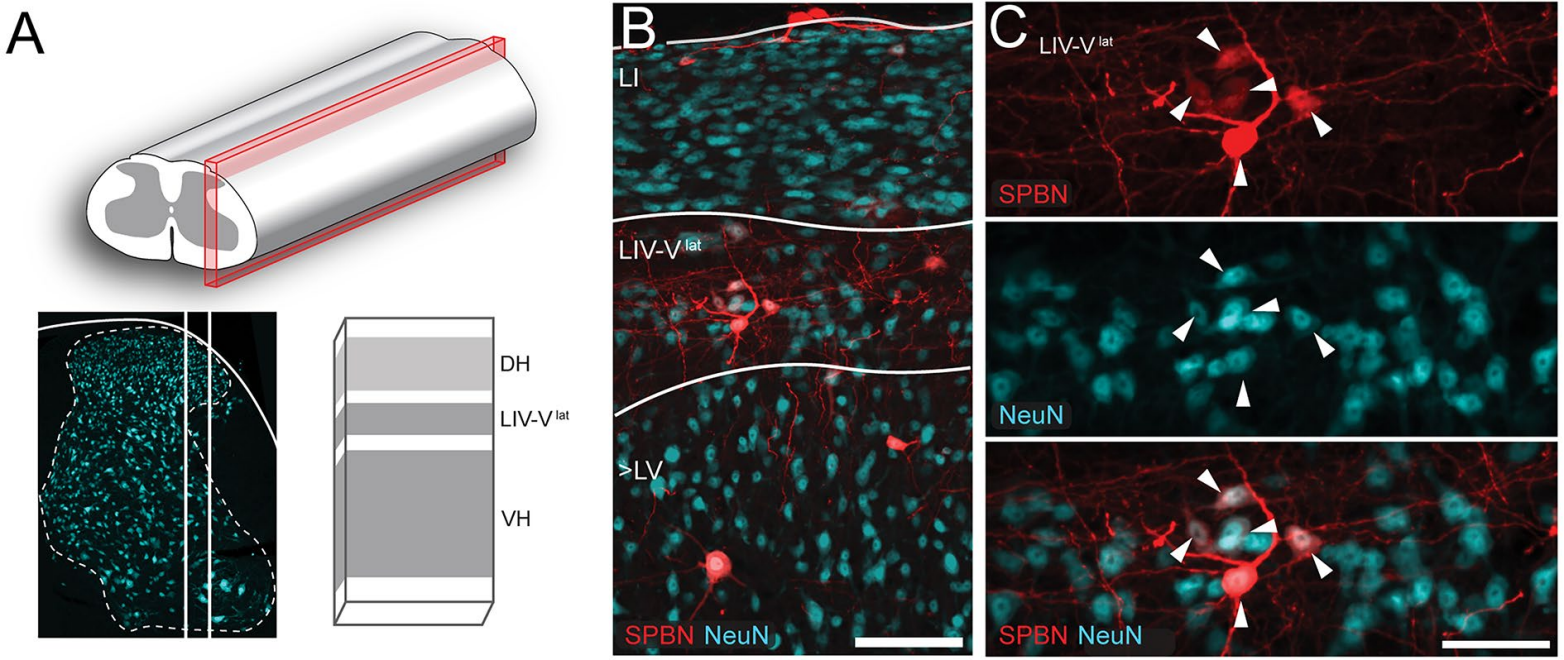
LV^lat^ SPBNs occupy lateral lamina V and the lateral grey matter. (**A**) images outline sagittal sectioning strategy and landmarks to identify the LV^Lat^ cell column. The red box overlaid on the spinal cord (top) highlights the mediolateral positioning of the sagittal section used for analysis. A fluorescent image labelled for NeuN (lower, left) shows the corresponding neuronal packing within the reticulated area of lateral LV through this section and emphasises the paucity of neuronal labelling in the area where lateral fasciculi lies. The schematic (lower right) summarises the resulting appearance of grey (neuropil) white matter banding in sagittal section. (**B**) fluorescent image labelled for NeuN and AAV9-RFP transduced SPBNs shows the dense neuronal packing within the SDH (upper), the emergence of the fasciculi within the LIV/V^lat^ area (middle), and then a transition into the less populated but consistently packed intermediate and ventral spinal areas (lower). The typical locations of superficial SPBNs (LI) can been seen at the dorsal section edge, LV^lat^ SPBNs are in the reticulated border, and some deeper SPBNs (> LV) are also present. (**C**) higher magnification image (from **B**) emphasises sparse neuronal packing and the relative low proportion of AAV9-RFP labelled LV^lat^ SPBNs. Scale: A: 100 μm, and B: 50 μm. Abbreviations: DH: Dorsal Horn. LIV-V^lat^: the lateral region of Lamina IV-V, VH: Ventral Horn, SPBN: Spinoparabrachial Neurons.

Across all labelling approaches, the LV^lat^ SPBN population represented a discrete cell column positioned in the deep dorsal horn, concentrated in the lateral reticulated areas where parallel fasciculi and a narrow band of dorsal grey matter exist. The proportion of LV^lat^ SPBNs in this region was assessed by comparing NeuN expression with RFP-labelling from unilateral PBN injections of AAV9-Cb7-Cl-mCherry (AAV-RFP, *n* = 3, Fig. 3). This analysis showed a relatively low overall density of NeuN labelled profiles, with AAV9-labelled SPBNs constituting approximately 12% of the LV^lat^ cell column (Fig. 3: Animal 1: *n* = 48/338, 13.6 ± 0.02%, Animal 2: *n* = 35/273, 11.6 ± 0.04%, Animal 3: *n* = 74/688, 11.0 ± 0.02%). Using the relative proportion of AAV9/rAAV2 overlap from the above analysis, AAV9-RFP counts were extrapolated to estimate total LV^Lat^ SPBN proportions of 23.37% and 21.25% in the LV^Lat^ region of each animal.

**Fig. 4:**
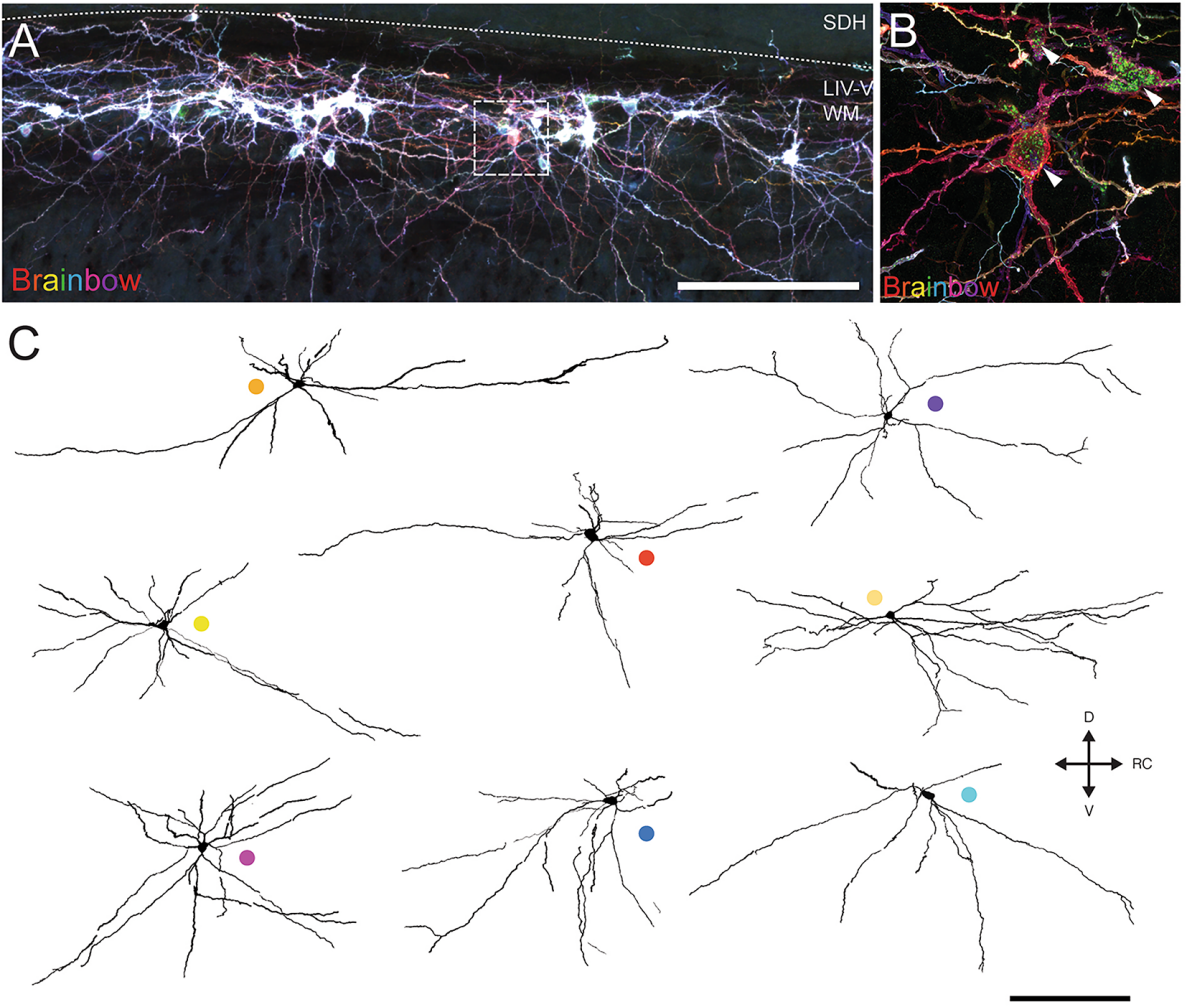
Analysis of Brainbow labelled LV^Lat^ SPBNs. (**A**) Image shows a lateral sagittal section containing intersectional viral labelling of LV^Lat^ SPBNs with brainbow. Note a dense column of cells in the lateral LIV-V region, including strong labelling of somatodendritic profiles. (**B**) A higher magnification image (denoted by box in **A**) showing clear colour separation and clearly distinguished dendritic arbors within the labelled LV^Lat^ population. (**C**) Images are partial reconstructions of a sample of LV^Lat^ SPBNs with distinct Brainbow colour labelling, mapped across 4 serial 50 μm sagittal sections (total mediolateral reconstruction over 200 μm). Coloured circles identify specific neurons for subsequently presented data.

Dense dendritic RFP labelling of LV^lat^ SPBNs in the above tissue precluded detailed analysis of individual neuron morphology. This was addressed in 4 additional animals that received an rAAV2-Cre injection in the right PBN and an intraspinal injection of AAV9-Flex-Brainbow to the left spinal dorsal horn to produce intersectional multi-colour Brainbow labelling of LV^lat^ SPBNs for reconstruction of somatodendritic dendritic morphology (Fig. 4). In our trials of rAAV-Cre, Cre-dependent labelling of LV^lat^ SPBNs, assessed using rAAV2-CreGFP, broadly reflected the expression achieved with other rAAV2 constructs (Supplementary Fig. 1A–C). Specifically, CreGFP expressing neurons were concentrated in the LV lateral cell column along with small numbers in LI, LVI/LVII and LX (Supplementary Fig. 1A-C). However, in subsequent intersectional experiments, the resulting CreGFP expression within the spinal cord and neurons that underwent recombination with Cre dependent Brainbow viral constructs (Supplementary Fig. 1Di-Dv) also revealed cells labelled within Lamina I, Antenna cells in LIII/LIV, LV medial neurons, and LX neurons. Despite this, the LV^lat^ neuron somatodentritic morphology could be confidently analysed. A total of 94 brainbow labelled LV^lat^ SPBNs were identified contralateral to PBN injection. Interestingly, in 2 animals, a small population of SPBNs were located ipsilateral to PBN injection, also positioned in LV^lat^ (*n* = 8). Overall, the number of LV^lat^ SPBNs labelled per animal varied (Animal 1: *n* = 69; Animal 2: *n* = 1; Animal 3: *n* = 16; and Animal 4: *n* = 8, respectively), however, the overall distribution patterns were strongly overlapping. The maximum soma cross sectional area of LV^lat^ SPBNs was similar across three animals but larger in the remaining one (Maximum cross-sectional area: Animals 1, 2, 4 vs. 3 (A1: 178.4 ± 39 mm^2^, A2: 189 ± 0 mm^2^, A4: 187.3 ± 89 mm^2^ vs. A3:242.2 ± 69 mm^2^; ANOVA: *p* = 0.000). The number of primary dendrites was consistent across all animals with a population average of 5.3 ± 1.2 dendrites (Primary dendrites = A1: 5.2 ± 1.2 vs. A2: 5 ± 0 vs. A3: 5.5 ± 1.1 vs. A4 5.62 ± 1.6; ANOVA, *p* = 0.717). Close inspection of the primary dendrite relationship with LV^lat^ SPBN somas suggested most of these neurons had multipolar morphology (primary dendrites > 4: 90/94; 95.7%), some exhibiting as many as 7 or 8 dendrites (*n* = 16 and *n* = 3, respectively). The remaining LV^lat^ SPBNs exhibited pyramidal morphology with 3 primary dendrites (*n* = 4/94: 4.3%).

**Fig. 5:**
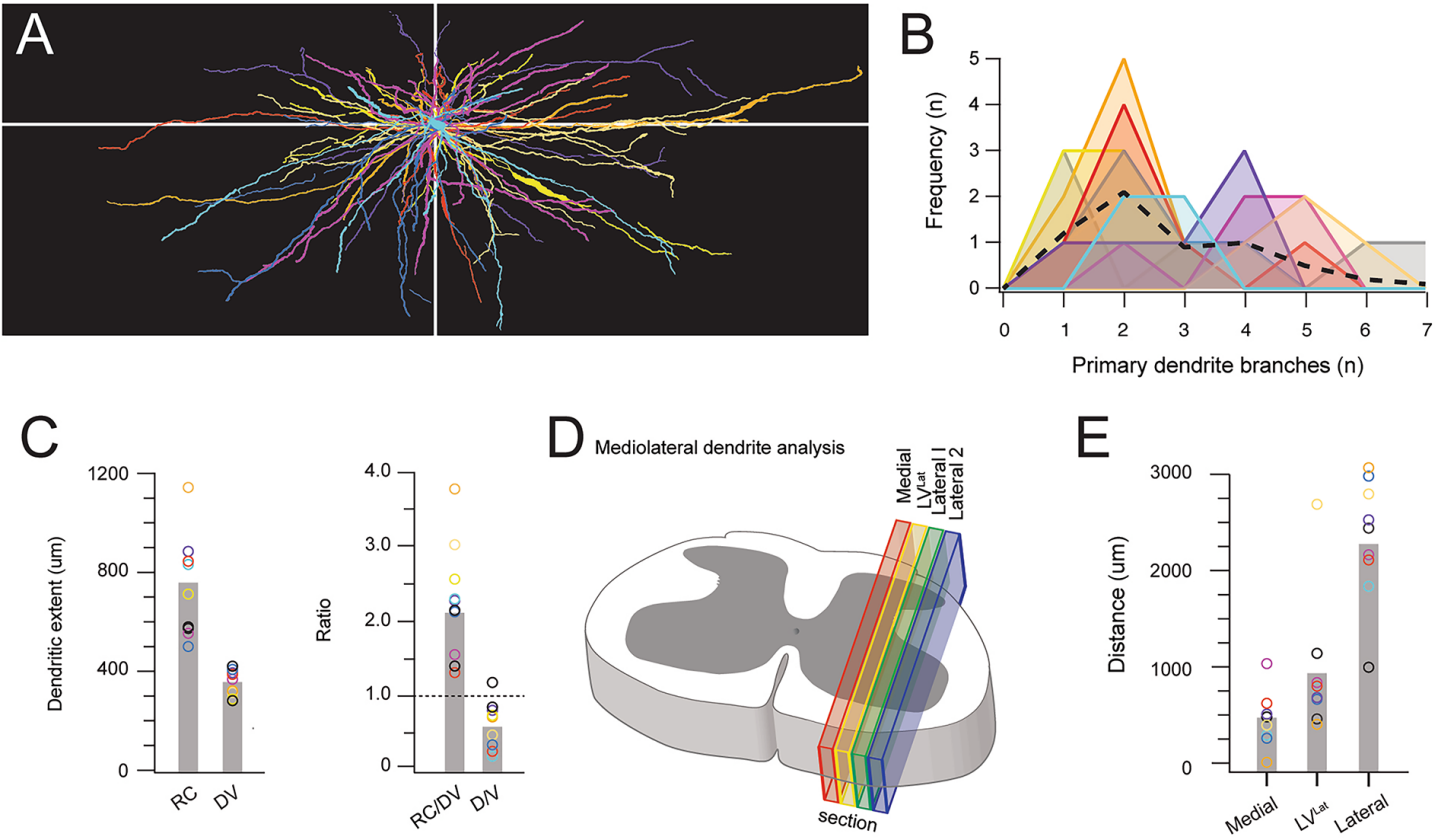
Analysis of Brainbow labelled LV^Lat^ SPBNs. (**A**) Overlaid LV^Lat^ SPBN reconstructions (from Fig. 4C) aligned to each cell’s soma. Note the consistent orientation of dendritic fields with wider rostrocaudal dendrite distributions (versus dorsoventral spread), and ventral dendrites spanning a greater area than dorsal dendrites. (**B**) Histogram shows the distribution of primary LV^Lat^ SPBN dendrite branches (per dendrite), showing dendrites branched infrequently despite spanning extended distances. Dashed line shows group mean. (**C**) Group data plots compare LV^Lat^ SPBN dendritic extent in the rostrocaudal (RC), and dorsoventral (DV) planes (left). Plots also summarise of RC/DV and D/V ratios (right). Dashed line denotes a ratio of 1, indicating no bias to distributions between compared planes. LV^Lat^ dendrites extended on average twice the distance in the RC plane (RC/DV > 2), whereas there was a bias for dendrites to extend further in the ventral than dorsal plane (D/V < 1.0). (**D**) Schematic summarises analysis of LV^Lat^ SPBN dendritic distribution in the mediolateral plane across serial sagittal sections. Sections were defined as LV^Lat^ when they contained the SPBN soma, and then the next slice immediately medial, and two immediately lateral (Lateral 1 & Lateral 2) were also included. The total length of LV^Lat^ SPBN dendrite included in each section was then calculated (Medial, LV^Lat^, Lateral, and Lateral 2). (**E**) Group data plots compare dendritic length in medial, LV^Lat^, and Lateral (Lateral 1 and Lateral 2 summed) sections. Note LV^Lat^ SPBN dendrites exhibited a distinctly lateral dendrite distribution. Scale bar: in A & C: 200 μm, B: 20 μm. Abbreviations: RC: rostrocaudal, DV: Dorsoventral, TDL: Total dendritic length, SDH: Superficial Dorsal Horn, LIV-V WM: Rexed lamina IV-V white matter.

A detailed morphological analysis of was undertaken for 10 LV^Lat^ SPBNs, reconstructed in the sagittal plane over 4 consecutive 50 μm sections using the soma as the starting point (Figs. 4 and 5). All sections were assessed for dendrite and followed maximally in each section. This highlighted a lack of branching in the medial plane, and extensions into the lateral spinal cord. Despite exhibiting a substantial total dendritic length (Range: 2585–5859 μm, Average: 3684 ± 875 μm), these cells had relatively simple dendritic profiles with primary dendrites exhibiting few branching points (Fig. 5B: Branching points: *n* = 54 dendrites: range: 1–8 total branches, Average: 2.8 ± 1.6 branches/dendrite; Primary dendrite length: range: 40–2503 μm, Average: 573 ± 445 μm). When comparing the territories occupied by LV^Lat^ SPBN dendrites, they were principally oriented in the rostrocaudal compared to dorsoventral axis (RC: 735 ± 198 μm vs. DV: 342 ± 55 μm; RC/DV ratio = 2.2 ± 0.8), averaging twice the territory in the rostrocaudal versus dorsoventral plane (Fig. 5C). Within the dorsoventral axis, there few dorsally oriented dendrites that extended outside the LV^Lat^ cell column, yielding a bias towards ventrally projecting dendrites (D:116 ± 49 μm vs. V: 228 ± 64 μm; D/V ratio = 0.57 ± 0.3). To better reflect the dendritic bias, we reported the total dendrite length in each section across the mediolateral plane (Fig. 5D). This confirmed a bias for LV^Lat^ SPBNs to have more dendrites towards the lateral white matter, with most dendritic profiles remaining lateral to the cell soma (Fig. 5E: M: 505 ± 220 μm vs. L: 3219 ± 902 μm; M/L ratio = 0.17 ± 0.09). Further, comparison of the proportion of dendritic reconstructions across sections showed that only 13% occupied territory medial to the cell somas, 25% remained within the LV^Lat^ cell column, 37% was in the lateral white matter adjacent to the LV^Lat^ cell column, and 25% in the most lateral white matter region.

### LVlat SPBN axons have diverse territories

**Fig. 6:**
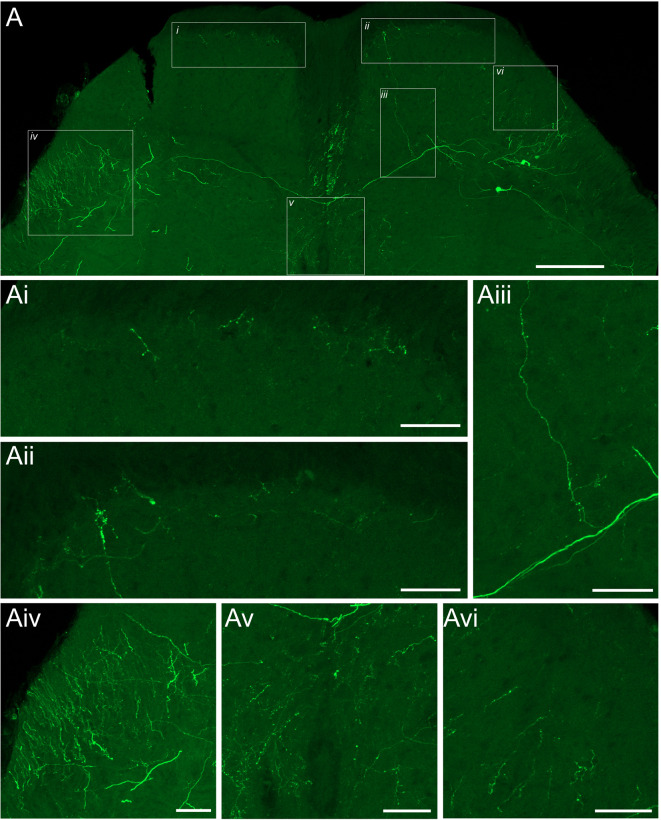
Axons of LV^;at^ neurons have diverse territories. (**A**) Fluorescent image of a single spinal section with LV^lat^ SPBNs labelled from an injection to the PBN. Areas are highlighted where axonal profiles are observed. **Ai-vi**, are higher magnification images from areas depicted in A. **Ai**, depicts axon terminals in the most superficial laminae of the dorsal horn ipsilateral to the injection and **Aii**, axon terminals contralateral to the injection. **Aiii**, image shows some consistent diameter profiles oriented in the mediolateral plane, highlighting that one of these axons gives rise to a collateral branch which then ascends into the most superficial laminae, with terminals and boutons visible. The terminal structures from **Aiii**, can be seen in **Aii. Aiv**, image depicts a bundle of sectioned axons, likely the main ascending pathway for LV^lat^ as it was common to see axons from within LV^lat^ entering this area. **Av**, image depicts dense labelling around the central canal and LX. A**vi**, image depicts axon profiles within the dorsal horn immediately dorsal to LV^lat^. Scale: A: 200 μm, Ai-Avi: 50 μm.

Tissue from rAAV2-GFP labelling of LV^Lat^ SBPNs was used to assess axonal territories (Fig. 6A). Labelled axons could be observed crossing the mediolateral DDH, entering the posterior commissure before crossing into the contralateral DH, with some clear examples passing into the contralateral LV^lat^. Axonal labelling was also within the dorsal funiculus. Axon terminals were often observed in both the ipsilateral and contralateral DH (Fig. 6Ai, Aii), though in most cases we were not able to be traced back to a specific LV^lat^ soma. Despite this, there were clear examples of axons branching from a commissural axon and ascending to the most superficial laminae (Fig. 6Aiii). The most densely labelled fibre tract, and presumed ascending pathway was located ipsilateral to the PBN injection within the dorsolateral funiculus, with axons often seen entering into this area from the LV^lat^ region (Fig. 6Aiv). In addition, axon terminals were commonly observed within medial Lamina V, Lamina X (Fig. 6Av) and within the grey matter of the lateral SDH (Fig. 6Avi). These axon termination patterns provide the potential for local spinal signalling from LV^lat^ collaterals in several spinal circuits.

### LVlat SPBN photostimulation recruits other spinal populations

**Fig. 7:**
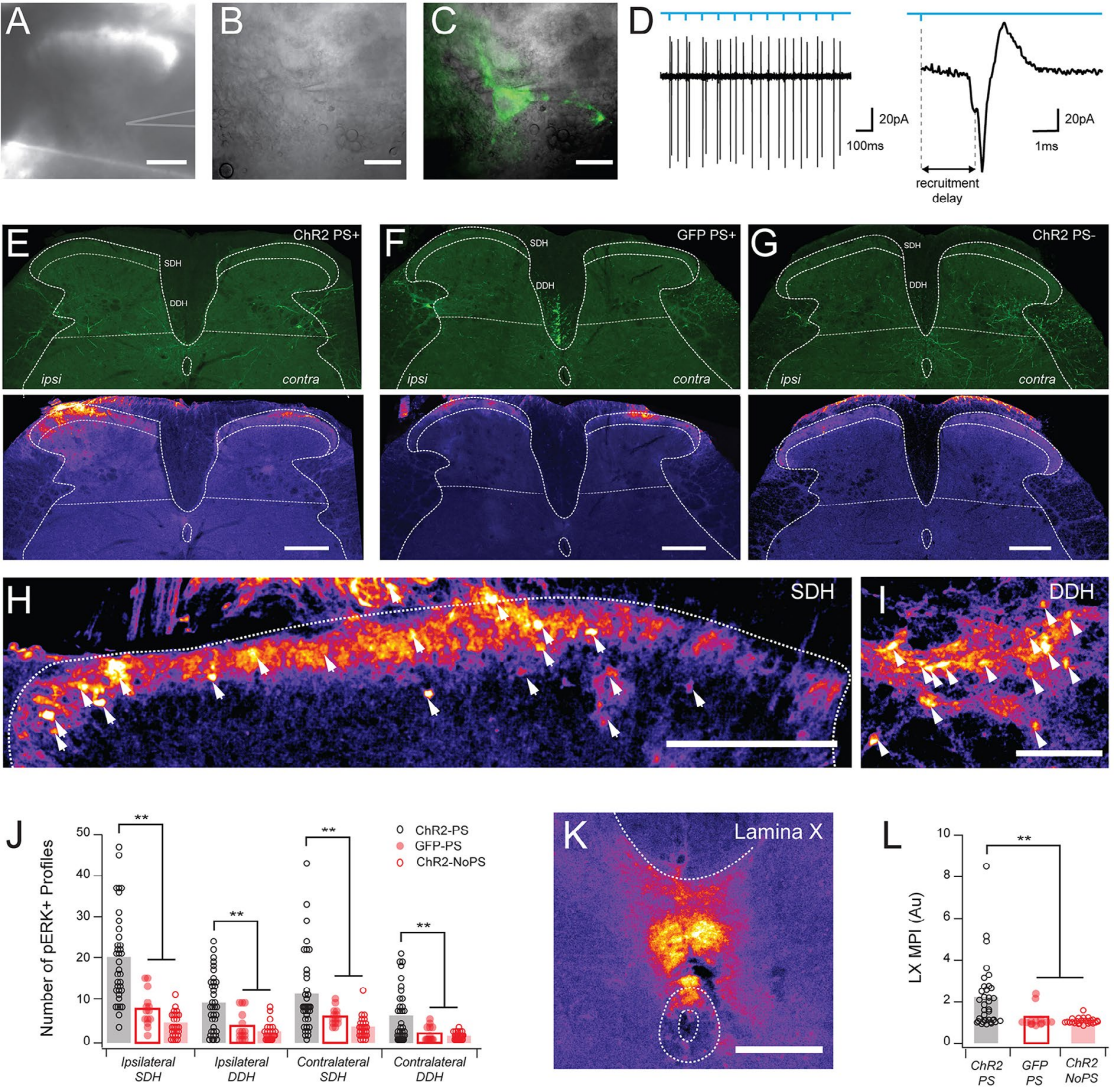
In vivo SPBNs photostimulation produces pERK expression in the spinal cord. (**A**) Bright field image of a spinal cord slice with recording pipette highlighted. (**B**,**C**) Higher magnification images (40x) show the recording pipette targets a ChR2-positive SPBN in the lateral part of lamina V in the deep dorsal horn. (**D**) left trace shows an on-cell voltage clamp recording from a ChR2-expressing SPBN during 10 Hz photostimulation (upper blue line − 1ms pulses, 470 nm). Note several extracellular action potentials corresponding to photostimulation timing. Right trace shows one photostimulation evoked AP response with the recruitment delay denoted (dashed line). **(E–G**) Upper image panels show transverse spinal cord sections from 3 experimental paradigms: LV^Lat^ SPBN ChR2 expression and photostimulation (ChR2 PS+); LV^Lat^ SPBN GFP expression and photostimulation (GFP PS+), and LVLat SPBN ChR2 expression without photostimulation (ChR2 PS-). Images below show corresponding pERK expression produced by each paradigm. pERK pixel intensity pseudocolored purple to yellow for low to high pERK expression. In ChR2 PS + animals, photostimulation produced an intense band of pERK in the ipsilateral superficial dorsal horn, and some pERK in the deep dorsal horn. In GFP PS + animals, photostimulation produced negligible pERK expression, controlling for activation due to light exposure alone. In ChR2 PS- animals, there was limited pERK expression controlling for the spinal cord exposure procedures. (**H**,**I**) higher magnification images of the SDH and DDH in a ChR2 PS + animal showing the punctate pERK + neuronal profiles. (**J**) group data plot summarises counts of pERK + profiles in ChR2:PS+, GFP: PS+, and ChR2:PS- paradigms. There were significantly more pERK profiles in ChR2:PS + than in GFP: PS+, and ChR2:PS- trials in all regions assessed. (**K**) high magnification image in the Lamina X region highlights substantial pERK signal, consistent with a dense plexus of SPBN axons decussating through this region. (**L**) group data plot shows significantly more pERK signal in ChR2:PS + trials (left), compared to the GFP: PS + and ChR2:PS- controls (middle and left, respectively). Scales: A, E-G: 200 μm; H:100 μm; I, K: 50 μm; B-C: 20 μm.

To facilitate a search for postsynaptic collateral connections, LV^Lat^ SPBNs were optogenetically stimulated and the expression of the activity marker, phosphorylated extracellular receptor kinases (pERK) was assessed in the spinal cord. First, efficacious rAAV2-mediated transduction with ChR2 and photostimulation responses in LV^Lat^ SPBNs were confirmed using targeted patch clamp recordings in spinal cord slices (Fig. 7A–C). On-cell recordings avoided altering cell excitability as has previously been demonstrated for the whole-cell configuration^38^. All LV^Lat^ SPBN tested exhibited robust AP discharge in response to photostimulation (*n* = 3, Fig. 7D), confirming the model was adequate to assess connectivity. Next, mice with unilateral rAAV2-ChR2 virus injections (*n* = 9) received partial unilateral laminectomies and spinal photostimulation was applied under anaesthesia to elicit phosphorylated ERK expression in any activated neurons. Three paradigms were assessed to control for pERK expression unrelated to SPBN photostimulation: (1) rAAV2-mediated ChR2 expression with photostimulation as the active trial (ChR2:PS+; *n* = 4); (2) rAAV2-mediated ChR2 expression without photostimulation (ChR2:PS- ;*n* = 3) as a control for the spinal exposure and laminectomy procedures; and (3) rAAV2-mediated GFP expression with photostimulation as a control for spinal photostimulation (GFP: PS+;*n* = 2). At the conclusion of each paradigm animals were perfused and tissue processed to identify pERK-positive profiles (Fig. 7E–G). A total of 77 transverse spinal sections were analysed, 36 for the ChR2:PS + photostimulation trials, and 41 for the ChR2:PS- and GFP: PS + controls, with 7–10 sections assessed per animal (summarised in Table 1). A total of 2120 pERK positive profiles were identified across both ipsilateral and contralateral dorsal horns (relative to photostimulation). There were significantly more pERK profiles across the superficial (Fig. 7H) and deep DH (Fig. 7I) on both the ipsilateral and contralateral spinal cords of the ChR2:PS + trial animals, compared to the either control (SDH Ipsi: 7.5 ± 4.1 vs. 20.2 ± 11.1 neurons; *n* = 50; z = 5.856, *p* = 0.000**, ChR2:PS- vs. ChR2:PS+), (3.67 ± 2.8 vs. 20.11 ± 11.1 neurons; *n* = 63, z = 4.452, *p* = 0.000**, GFP: PS- vs. ChR2:PS+), and a comparison of all 3 conditions also highlighted the significantly increased pERK profiles in the active trial (ChR2:PS- vs. GFP: PS + vs. ChR2:PS+: 20.1 ± 11.1 vs. 7.5 ± 4 vs. 4.1 ± 3.1 neurons; ANOVA: 32.684, *n* = 77, *P* = 0.000). These results support the hypothesis that SPBN axon collateral activation can engage spinal circuits.

**Table 1:** Comparison of pERK + profile counts for active photostimulation trial (ChR2 PS+), and controls trials for generic photostimulation responses (GFP PS+), and surgical preparation responses (ChR2 PS-).

Animals/sections analysed	ChR2 PS + trial	GFP PS + control	ChR2 PS- control	Statistics
	*n* = 4/36	*n* = 2/14	*n* = 3/27	
Ipsilateral SDH	20.11 ± 11.1 (3–47) PS+**> GFP > PS-	7.5 ± 4.1 (1–15) Similar to PS-	4.1 ± 3.1 (0–11) Similar to GFP	ANOVA: 32.684, *p* = 0.000, *N* = 77
Ipsilateral DDH	9.5 ± 6.4 (0–24) PS+**> GFP > PS-	3.29 ± 3.4 (0–9) Similar to PS-	1.96 ± 2.1 (0–8) Similar to GFP	ANOVA: 21.247, *p* = 0.000, *N* = 77
Contralateral SDH	11.2 ± 9.6 (0–43) PS+**> GFP > PS-	5.6 ± 2 (3–10) Similar to PS-	3.2 ± 2.5 (0–12) Similar to GFP	ANOVA: 10.859, *P* = 0.000, *N* = 77
Contralateral DDH	6 ± 6.1 (0–21) PS+**> GFP > PS-	1.4 ± 1.8 (0–5) Similar to PS-	0.9 ± 0.8 (0–3) Similar to GFP	ANOVA: 12.456. *P* = 0.000, *N* = 77

Regarding the distribution of the photostimulation-activated cells, the greatest number of pERK positive profiles were detected in the SDH ipsilateral to photostimulation (Table 1; Fig. 7J), and there were significantly more pERK positive profiles per section in the ChR2:PS + versus controls (SDH-Ipsi: 20.1 ± 11.1, *p* = 0.000; Fig. 7E,D). Surprisingly, the contralateral SDH had the next most pERK profiles (SDH-Contra: 11.2 ± 9.6, *p* = 0.000), followed by the ipsilateral DDH (DDH-Ipsi: 9.5 ± 6.4; *p* = 0.000; Fig. 7E,I) and the contralateral DDH (DDH-Contra: 6.0 ± 6.1; *p* = 0.000; Fig. 7J). This indicates that photostimulation of LV^lat^ SPBNs recruited neurons across the spinal cord, well above non-specific pERK activity induced by the surgical preparation and spinal cord light exposure alone. Given PBN injection of rAAV2 also transduced LV^Lat^ SPBN terminals in LX around the central canal, we compared pERK labelling between photostimulation and control trials in this area (Fig. 7K,L). This analysis showed a significant increase in pERK fluorescence in the ChR2:PS + trial to controls (2.1 ± 1.5 vs. 1.2 ± 0.45 vs. 1.1 ± 0.14 arbitrary units, ANOVA: 7.711, *P* = 0.001, *N* = 76; Tukeys HSD: ChR2:PS+** > GFP: PS+, ChR2:PS-). Together, these data further support the hypothesis that as LV^lat^ SPBNs are activated local axons collaterals can mediate recruitment of other DH populations across the superficial and deep dorsal horns bilaterally.

### LVlat SPBNs provide synaptic input into superficial dorsal horn circuits

**Fig. 8:**
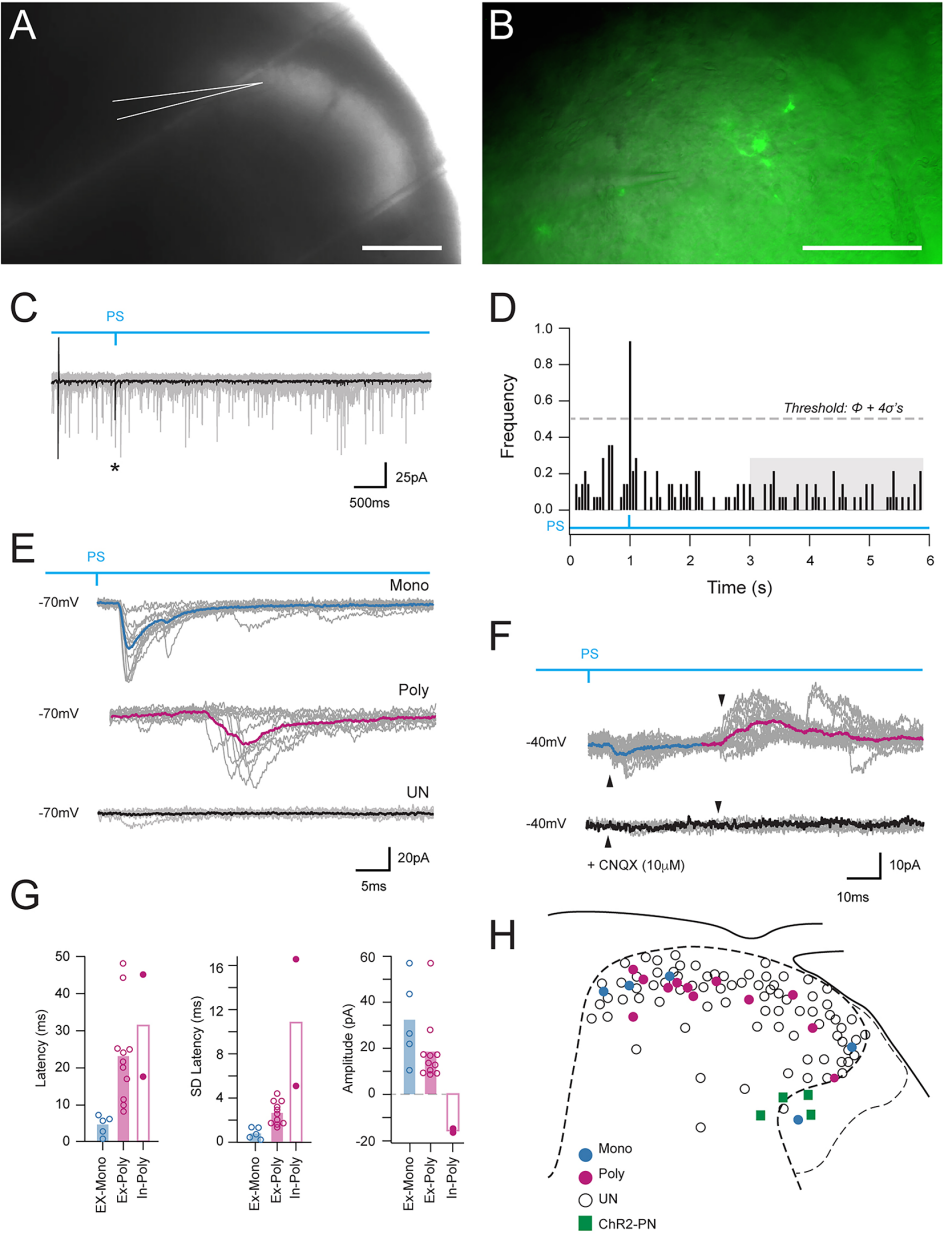
Optically evoked postsynaptic currents (oPSCs) recorded in dorsal horn neurons during LV^Lat^ SPBN photostimulation. (**A**) A brightfield image of a spinal cord slice with recording pipette highlighted. (**B**) A higher magnification image depicting an established recording near axonal terminals in the SDH. (**C**) Summary of photostimulation oPSC response analysis. Overlaid voltage clamp traces (top, 10 trials in grey; average response in black) exhibit ongoing spontaneous postsynaptic currents (sPSCs). Brief photostimulation is applied (blue line, 1ms pulse at 1s) causing a large optically evoked postsynaptic current (oPSC) apparent in the averaged trace. (**D**) Histogram (below) shows PSC frequency (50ms epochs), with the last 3 s of data used to calculate background sPSC frequency (grey shaded box). Note the peak in PSC frequency histogram immediately following photostimulation, confirming an oEPSC response. Neurons were deemed to receive a photostimulation evoked input if there was a frequency peak following photostimulation above spontaneous background frequency (mean + 4SD, grey-dashed line). (**E**) representative voltage clamp traces (10 trials with the average overlaid, holding potential: -70mV) show the three different types of LV^Lat^ SPBN photostimulation responses including: oEPSCs at short-latency, reflecting monosynaptic SPBN input (upper traces); oEPSCs at longer latency, consistent with polysynaptic SPBN circuit activation (middle traces); or no oEPSC response (lower traces). Top blue line denotes photostimulation. (**F**) example recording with holding potential adjusted to -40mV revealed a short latency excitatory (inward) current and longer latency inhibitory (outward) current, with both abolished by CNQX (lower trace). (**G**) group data plots compare oEPSC response latency, jitter (latency SD), and amplitude for monosynaptic and polysynaptic excitatory responses (Ex-Mono and Ex, Poly, respectively) and polysynaptic inhibitory responses (In-Poly). Note short latency and low jitter of monosynaptic connections, contrasted by longer latency and higher jitter in polysynaptic connections. Amplitudes varied across both connection types; however, negative amplitudes (inhibitory input) were only observed in polysynaptic responses. (**H**) summary map presents recording locations of all neurons sampled during photostimulation recording experiments (monosynaptic connection = blue, polysynaptic connection = red, no connection = open circle, and recorded SPBNs = green). Note most connections were identified in the superficial DH. Scale bar: A:200 μm, B: 50 μm.

Considering the substantial activation of superficial DH neurons by LV^lat^ SPBN photostimulation, we next sought to test for specific connectivity and the circuits underlying this observation. Channelrhodopsin-2 assisted circuit mapping (CRACM) of LV^lat^ SPBN axon collateral inputs was undertaken in transverse spinal cord slices (Fig. 8A, *n* = 16 animals, *n* = 80 slices, *n* = 106 neurons) with brief (1ms) whole field photostimulation applied while patch clamp recordings were made from unidentified DH neurons near fluorescent axon terminals (Fig. 8B). Given many LV^Lat^ SPBN axons were potentially severed in spinal slice preparation, 4-AP (200mM) was routinely added to enhance synaptic release probability. This enhanced our ability to resolve monosynaptic and polysynaptic circuitry engaged by LV^Lat^ SPBNs. A criterion was used for identification of input arising from LV^Lat^ SPBN axon collateral activation, where putative optically evoked postsynaptic currents (oPSCs) needed to occur at relatively short latency following photostimulation in multiple trials and exceed the mean background instantaneous sPSC frequency ± 4SD (Fig. 8C,D; see Methods). In addition, a threshold latency of 8ms was used to differentiate monosynaptic and polysynaptic responses (monosynaptic oPSC latency < 8ms; polysynaptic > 8ms). A total of 17 neurons (*n* = 17/106, 16.0%) met this criterion for input derived from local SPBN axon collaterals (Fig. 8E). Of these inputs, 5/18 (27.7%) were deemed monosynaptic and 13/18 (72.3%) polysynaptic. Within the polysynaptic responses, 11 were excitatory inward currents when recorded in voltage clamp (holding potential − 70mV), and 2 were inhibitory outward currents (Fig. 8F; holding potential − 40mV). Significant differences in the latency and jitter (SD of latency) of photostimulation evoked inputs (Fig. 8G) were consistent with the original assignment of putative monosynaptic excitatory connections and polysynaptic excitatory or inhibitory connections (latency: 4.65 ± 2.6 ms vs. 23.59 ± 13.7 ms vs. 31.39 ± 19.4 ms; *P* = 0.02 and jitter: 0.8 ± 0.5 ms vs. 2.63 ± 1.0 ms vs. 11.0 ± 5.9 ms, *P* = 0.00). Comparison of the amplitude of excitatory monosynaptic and polysynaptic connections did not differ (-31.85 ± 18 pA vs. -18.19 ± 14.0 pA, *P* = 0.15), while polysynaptic inhibitory connection amplitude was 15.59 ± 1.2 pA. One recording contained a monosynaptic excitatory (inward) current and polysynaptic inhibitory (outward) current (n = 1; Fig. 8F), while the remaining recordings contained a single input (n = 16). Thus, 18 total connections were observed from recordings in 17 neurons. When recorded in current clamp, photostimulation evoked inputs remained subthreshold (i.e., did not evoke AP discharge). A subset of recordings was also assessed following bath applied AMPA/Kainate receptor antagonist, 6-cyano-7-nitroquinoxaline-2,3-dione (Fig. 8F) CNQX; *n* = 5), which abolished all photostimulation-evoked responses confirming their dependence on glutamatergic signalling. The dorsoventral and mediolateral position of recorded neurons receiving LV^lat^ SPBN-derived input was mapped, highlighting the location of responsive neurons concentrated in the superficial DH and distributed across the mediolateral dimensions (Fig. 8H). Together, these findings show for the first time that axon collaterals from a population of LV^lat^ SPBNs activate a range of circuits likely to influence spinal nociceptive processing.

### Electrophysiological characteristics of cells receiving LVlat SPBN-derived input

**Fig. 9:**
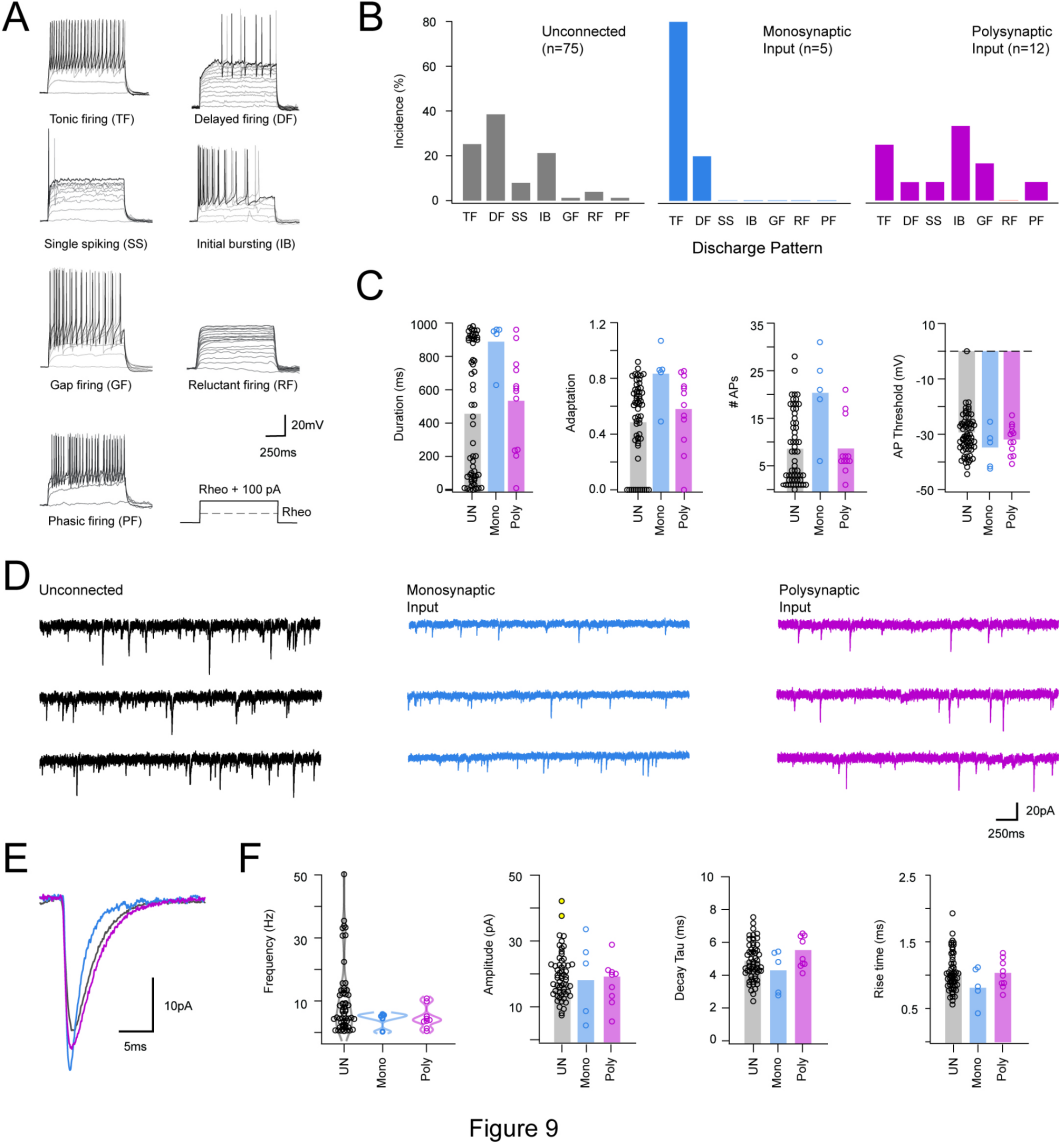
Electrophysiological profile of neurons receiving photostimulation-evoked LV^Lat^ SPBN axon collateral evoked input. (**A**) Overlaid traces show AP discharge patterns evoked by depolarizing current step injections in unidentified neurons sampled in photostimulation mapping experiments. Responses were classified as: tonic firing (TF), delayed firing (DF), single spiking (SS), initial bursting (IB), gap firing (GF), reluctant firing (RF), and phasic firing (PF), based on previous descriptions^12,36,42^. (**B**) Group data plots compare the incidence of different discharge patterns across neurons that were; unconnected (UN), received monosynaptic LV^Lat^ SPBN input (mono-oPSCs), or received polysynaptic LV^Lat^ SPBN input (poly-oPSCs). Note TF responses were dominant in neurons receiving monosynaptic input but less common in neurons received Poly-oPSC input or UN. (**C**) group data compare selected features of repetitive AP spiking responses including, discharge duration, spike frequency adaptation, number of action potentials discharged, and action potential threshold for the rheobase + 100pA current step response. Note neurons receiving mono-oPSC input exhibited greater repetitive firing, increased discharge duration, less frequency adaptation, but similar AP thresholds. (**D**) traces showing 10s of representative sEPSC activity in unconnected, mono-oPSC, and poly-oPSC responses to LV^Lat^ SPBN photostimulation. (**E**) overlaid traces compare averaged sEPSCs (from traces in D) in unconnected, mono-oPSC, and poly-oPSC groups. (**F**) group data compares sEPSC frequency, amplitude, decay time constant (Tau), and rise time between unconnected, mono-oPSC, and poly-oPSC recordings. sEPSC frequency was lower in the mono-oPSC and poly-oPSC groups compared to UN, but there were no differences in the average amplitudes, Tau, or rise times. Traces and data: black = UN, blue = mono-OPSC, magenta = poly-OPSC.

Based on the well-established literature associating electrophysiological features with neuron identity in the DH (e.g., excitatory vs. inhibitory)^39–41^ the electrophysiological characteristics of neurons that received LV^lat^ SPBN collateral input were assessed and compared (Fig. 9). This sample was separated into 3 populations: neurons with monosynaptic SPBN input (mono-oPSC); polysynaptic SPBN input (poly-oPSC); and neurons that did not exhibit connections (UN). First, baseline characteristics such as cell input resistance and membrane capacitance were similar across populations (UN vs. mono-oPSC vs. poly-oPSC; *n* = 83, 5, 12; R_in_ = 406.55 ± 195.9 MΩ vs. 330.60 ± 104.5 MΩ vs. 407.25 ± 241.7 MΩ, *P* = 0.71: C_m_ = 11.90 ± 6.4 pF vs. 12.78 ± 3.9 pF vs. 12.97 ± 12.4 pF, *P* = 0.86), as was the rheobase current required to evoked AP discharge and the AP threshold (rheobase: 57.68 ± 44 pA vs. 64 ± 76.7 pA vs. 49.23 ± 32.3 pA, *P* = 0.77; AP threshold: -29.34 ± 8.7 mV vs. -34.77 ± 7.2 mV vs. – 31.89 ± 5.3 mV, *P* = 0.26, UN vs. mono-oPSC vs. poly-oPSC; *n* = 70, 5, 12). Next, we report the incidence of action potential discharge patterns following depolarising current injections (Fig. 9A,B). From the small sample of mono-oPSCs and poly-oPSCs we did not meet the criteria for statistical comparison of the distributions. However, tonic firing (which is associated with inhibitory cell phenotype) was most common in neurons that received a monosynaptic SPBN input (TF: *n* = 4, 80%), while the remaining monosynaptically connected neuron exhibited a delayed firing response. Cells that received a polysynaptic input (*n* = 12) exhibited a wider range of discharge patterns including initial bursting (IB: *n* = 4, 33.3%), tonic firing (TF: *n* = 3, 25.0%), Gap firing (GF: *n* = 2, 16.7%), and single examples of delayed firing, phasic firing, and single spiking. Finally, neurons that did not receive LV^lat^ SPBN-derived input (*n* = 75) exhibited several discharge patterns typical of a random sample, including prevalent delayed firing (DF: *n* = 29, 38.7%), tonic firing (TF: *n* = 19, 25.3%), initial bursting (IB: *n* = 16, 21.3%), single spiking (SS: *n* = 6, 8.0%), reluctant firing (RF: *n* = 3, 4%), and individual examples of phasic firing and gap firing.

Consistent with a high prevalence of tonic firing in neurons with monosynaptic oEPSCs, AP discharge duration, discharge frequency, and spike frequency adaptation were significantly greater in this sample (Fig. 9C, mono-oPSC vs. poly-oPSC vs. UN: duration: 886.87 ± 144.9 ms vs. 533.45 ± 298.3 ms vs. 454.16 ± 391.6 ms, *P =* 0.000, Tukey HSD; mono-oPSC*< poly-oPSC < UN, *P* = 0.037, *n* = 5, 12, 60; AP frequency: 20.40 ± 9.3 Hz vs. 8.67 ± 6.0 Hz vs. 8.53 ± 7.5 Hz, *P* = 0.004, Tukeys HSD: mono-oPSC**> UN > poly-oPSC, *P =* 0.003, 0.010; Adaptation: 82.58 ± 20.9% vs. 57.76 ± 26.5% vs. 48.50 ± 29.9%, *P* = 0.037, Tukeys HSD: mono-oPSC*> UN, *P =* 0.036). In contrast, spike latency, mean interspike interval and spike height attenuation were similar across the sample (mono-oPSC vs. poly-oPSC vs. UN, *n* = 5, 12, 60 respectively; Latency to AP: 77.28 ± 119.7 ms vs. 39.88 ± 39.6 ms vs. 81.62 ± 162.8.0 ms, *P* = 0.68; interevent interval: 50.89 ± 43.7 ms vs. 56.03 ± 38.3 ms vs. 45.48 ± 47.1 ms, *P* = 0.76; attenuation: 77.63 ± 28% vs. 84.69 ± 110% vs. 59.18 ± 44%, *P* = 0.34). The incidence of different subthreshold voltage-activated currents was also in agreement with a high incidence of tonic firing in neurons receiving mono-oPCSs. Specifically, the incidence of I_A_ potassium currents that supress AP discharge was lowest in cells receiving mono-oPSCs (mono-oPSC vs. poly-oPSC vs. UN: *n* = 3, *n* = 6, *n* = 59; 33.3% vs. 66.7% vs. 64.4%).

Finally, the properties of spontaneous excitatory synaptic input were compared across the sample (Fig. 9D–F). sEPSC frequency was lower in neurons that received mono-oPSC input versus those lacking SPBN axon collateral mediated connections (mono- oPSC vs. poly-oPSC vs. UN; 4.24 ± 2.3 Hz vs. 4.92 ± 3.9 Hz vs. 9.79 ± 10.7 Hz, Welchs ANOVA, *P =* 0.009). There were no differences, however, in sEPSC amplitude (mono-oPSC vs. poly-oPSC vs. UN; *-*19.13 ± 6.9 pA *vs. -*18.1 ± 6.0 pA vs. -16.76 ± 6.4 pA, *P =* 0.62), time-course or charge (rise time: 0.81 ± 0.1 ms vs. 1.03 ± 0.1 ms vs. 1.02 ± 0.ms, *P =* 0.18; Tau: decay time constant: 4.26 ± 1.3 ms vs. 5.59 ± 1.1 ms vs. 4.86 ± 1.1 ms, *P =* 0.07; charge: 100.17 ± 36.0 pA.ms vs. 131.59 ± 71.1 pA.ms vs. 108.85 ± 47.4 pA.ms, *P =* 0.34. Together, this profile of tonic AP discharge, low expression of I_A_ currents, and low sEPSC frequency is consistent with monosynaptic LV^lat^ SPBN input from axon collaterals preferentially targeting inhibitory DH populations^39,42–44^.

## Discussion

This study provides evidence that, in addition a well-established role for SPBNs relaying sensory information to the brain, a population of SPBNs also influence local spinal circuits through axon collateral signalling. We establish this functional connectivity for LV^Lat^ SPBNs using AAV2-retro to transduce cells concentrated within the lateral part of the deep DH. The consistent and selective transduction of these cells, along with relatively uniform morphological characteristics, suggest LV^lat^ SPBNs constitute a novel distinct and potentially functionally discrete group of spinal projection neurons. In vivo spinal photostimulation of these cells under anaesthesia produced a widespread pattern of activation in the superficial and deep DH both ipsilateral and contralateral to the photostimulation area, as well as in the LX region around the central canal. Patch clamp recordings in spinal cord slices identified LV^lat^ SPBN axon collateral input onto superficial DH neurons with features of monosynaptic and polysynaptic circuit activation. Monosynaptic inputs were exclusively excitatory and targeted DH cells with electrophysiological characteristics of inhibitory interneurons, including high frequency AP discharge and low frequency excitatory drive. Polysynaptic input included excitatory and inhibitory connections and targeted cells with a more diverse range of electrophysiological properties suggesting recruitment of feedforward and feedback circuits. In summary, while the role of PNs in transmitting sensory information to higher brain regions for perception is well established^25^, the current data shows that a specific population of PNs (LV^lat^ SPBNs) share an efference copy of the information they relay to the brain, back into local spinal cord circuits.

Targeting a population of deep DH SPBNs in the current experiments comes from the serendipitous observation of serotype specific transduction by AAV2-retro-hsyn. This is consistent with other work drawing a similar conclusion, with Frezel et al. (2023) reporting AAV2-retro constructs produced expression in deep DH SPBNs and only few LI SPBNs^45^. Precedent for these observations has also been reported elsewhere with selective transduction of long-range descending brainstem and midbrain projections following spinal rAAV2 injections^46^. This work targeted noradrenergic Locus Coereleus neurons but only transduced a small Tyrosine hydroxylase negative population. Paralleling our study, substituting an AAV9 construct transduced a wider Locus Coereleus population including noradrenergic and non-noradrenergic populations^46^. Despite these observations, not all experiments using rAAV2 align with our results, and some work has concluded that AAV9 specifically captures DDH projection neurons and AAV2-retro is superior for capturing LI SPBNs^47^. Moreover, a recent publication using the AAV2-retro virus to study spinal projection neurons did report a population in lateral LV, but also labelled projection neurons in lamina I, II-IV, and medial LV^48^ complicating detailed LV^Lat^ SPBN analysis. Several factors may influence these differences including viral titre^49^, inclusion of cre-dependent transduction^46^, promoter differences^49^, and differential route of viral infection/uptake across specific serotypes^50^.

The uniform morphological characteristics resolved in our Brainbow analysis of LV^lat^ SPBNs, (Figs. 4 and 5), combined with a restricted spatial distribution in the DH, and the selectivity in rAAV2 transduction suggests these cells represent a novel and distinct population of spinal projection neurons. This is somewhat surprising with decades of work dedicated to characterising the ascending spinal sensory pathway, however, the relatively small nature of this population coupled with a distribution among the lateral white matter fasciculi may have obscured these cells without the benefit of selective labelling. Despite these factors, our isolation of the LV^Lat^ SPBN population coincides with two other reports that also highlight a population of projection neurons that share characteristics with our data. First, a viral transduction approach has been used to study ascending spinal outputs conveying nociceptive information to the brain and highlighted three distinct pathways, with one targeting the parabrachial nucleus via bilateral projections, and including cells in the lateral lamina V region^48^. Another study undertook single cell deep sequencing of Phox2a expressing cells, known to variably label spinal projection neurons in different laminae, and distinguished 5 projection neuron subpopulations on genetic expression profiles^51^. Importantly, one population, termed ALS4, were differentiated in the lateral LV region and presumably correspond to the LV^Lat^ cells described here. The ALS4 population could be distinguished by expression of Gpr88 and Errb4, showed relatively selective expression of Somatostatin, and a bilateral projection patten to the brainstem. Earlier work on the Phox2a-expressing population from the same group distinguished a morphologically distinct population of cells they termed non-antennae neurons and noted some of these cells were located in the lateral LV white matter and could be retrogradely labelled by CTB injection in PBN^52^. The term ascribed to these cells, non-antennae, referred to the lack of dorsally directed dendrites, also paralleled in our morphological reconstruction of rAAV2-labelled LV^lat^ SPBNs. Thus, converging evidence supports the view that LV^Lat^ cells do represent a discrete SPBN population.

Regarding function, no data has specifically assessed the behavioural outcome of LV^Lat^ SPBN activation, although viral tracing studies on bilateral spinoparabrachial projection neurons, including LV^Lat^ SPBNs, did undertake chemogenetic and optogenetic activation of transduced cells^48^. Both manipulations produced licking, flinching, and guarding responses consistent with nociceptive signalling, however, this activation would have included LI and other projection neuron populations as well as LV^Lat^ cells, and would be expected to mediate nociceptive signalling. Thus, it remains unclear how the LV^Lat^ population specifically contributed to these results. Some insight can also be drawn from the sources of synaptic input received by LV^Lat^ SPBNs. For example, Phox2a + non-antennae cells in the deep DH that likely overlap with the LV^Lat^ population, were reported to receive VGLUT1 + input (~ 12%) from myelinated A-low threshold mechanoreceptors and corticospinal projections. In addition, these cells received substantial VGLUT2 + input (55%), presumably from local circuit excitatory interneurons but very few inputs (4%) from Calcitonin Gene-Related Peptide + unmyelinated C fibres^52^. Together, this pattern of input is consistent with a role in processing touch related information for LV^lat^ SPBNs, whereas activation by nociceptive input appears most likely through polysynaptic connections mediated by excitatory interneurons. In support of a polysynaptic pathway, chemogenetic stimulation of an excitatory spinal interneuron population identified by the gastrin-releasing peptide receptor (GRPR), led to robust Fos expression in the lateral LV region^53^. Retrograde projection neuron labelling in these animals confirmed the greatest level of activation from GRPR interneurons occurred in LV^Lat^ SPBNs. Interestingly, the GRPR interneuron population has been shown to be activated by pruritic and noxious sensory input, and in vivo chemogenetic activation produces a phenotype consistent with itch and nociception^54^. These findings imply LV^Lat^ SPBNs receive and signal multiple sensory modalities.

Single cell sequencing that has highlighted the LV^Lat^ (ALS4) population express somatostatin also adds insights on potential functions^51^. Specifically, a number of previous studies have ablated spinal somatostatin-expressing cells and reported deficits in acute mechanical pain, attenuated pruritogen evoked scratching, and reductions to both dynamic and static mechanical allodynia in neuropathic and inflammatory models^55^. While these observations were attributed to loss of somatostatin-expressing interneurons, contributions by the parallel loss of the LV^Lat^ SPBN population are also likely. Finally, classical in vivo electrophysiological studies report on the sensory modalities that provide input to projection neurons in the deep DH, albeit not specifically identified as LV^Lat^ cells. Much of this work describes the deep projection neurons as exhibiting wide dynamic range properties, reflecting broad responses to both innocuous to noxious stimuli (for review see Wercberger and Basbaum, 2019 Curr Opin Physiol^37^). Further, one primate study distinguished deep DH projection neuron types based on sensory response profile and dendritic branching, describing a group that exhibited distinct ventrally biased dendrites and weak spiking responses to a range of cutaneous stimuli (i.e., wide dynamic range)^29^. Taken together, this literature suggests the LV^Lat^ SPBNs may be broadly tuned to a range of sensory modalities, however, future studies that selectively manipulate these cells will be required to fully resolve this issue.

To assess local spinal connectivity of LV^lat^ SPBNs, these cells were transduced with ChR2 via viral brainstem injection, however, this risks transducing other brainstem populations that provide descending spinal cord input, complicating interpretation of spinal photostimulation responses. Use of the rAAV2 viral serotype, selectively engineered with retrograde properties^56^, was chosen to mitigate this risk as previous work has reported adjoining brain regions with long range spinal projections such as Locus Coereleus are resistant to rAAV2 infection^46^. Our analysis also showed differences in rAAV2- and AAV9-mediated expression at brainstem injection sites compatible with rAAV2 retrograde specificity (Fig. 1). Recordings of photostimulation-evoked glutamatergic connections in our experiments are also consistent with responses derived from SPBNs as there is no evidence of a glutamatergic system originating from the brainstem and descending to terminate in the DH. Finally, no conductance’s typical of noradrenergic or serotonergic inputs were observed during CRACM recordings, which are the prominent output neurotransmitter systems of pontine and hindbrain descending systems^46,57^. Considered together, these observations support efficient and selective activation of LV^lat^ SPBNs during in vivo and in vitro spinal photostimulation.

The assays used to detect DH circuit activation by SPBN axon collaterals also warrant some consideration. For in vivo photostimulation experiments, phosphorylation of ERK identifies activation and has a signal peak of ~ 5 min post-stimulation^58^. This brings a risk that any contact with the animals during or following photostimulation (e.g., perfusion procedure) could also produce pERK expression. Controls for such additional activation sources only resolved low levels of pERK expression compared to our experimental ‘photostimulation’ trials, supporting the view that LV^lat^ SPBN axon collaterals do produce substantial DH activation. Whether this activation is due to direct local LV^Lat^ axon collateral signalling or mediated by ascending signals from LV^Lat^ SPBNs and subsequent descending activation cannot be differentiated under our experimental conditions. Nevertheless, the distribution of local LV^Lat^ axon collaterals in same regions where pERK activation was detected supports a contribution by direct local signalling. This interpretation is also supported by our slice-based electrophysiology, with CRACM detecting a range of photostimulation evoked responses that were dependent on glutamatergic signalling, albeit at relatively low frequency. This must be viewed against the likelihood that many axons are sectioned and truncated in the slice preparation, and thus inputs attributed to LV^lat^ SPBN collaterals reported here are likely an under-prediction. Regardless, our data now provides evidence that LV^lat^ SPBN outputs influence local spinal circuits via axon collaterals as well as serving their anatomically defined role of relaying sensory signals to higher brain regions.

Studies have noted local axon collaterals arising from spinal projection neurons for decades including spinoparabrachial neurons in cat^59^, rat^13–15^ and mouse^12^, ; spinothalamic neurons in cat^10,11,60^ and monkey^7^, and spinocervical neurons in cat^61–64^. While most studies simply note evidence for local axon collaterals, a more detailed quantitative characterisation has come from the work of Safronov and Szucs, classifying PN collaterals on distribution and trajectory into dorsal, lateral, ventral, mixed, and contralateral types^13,14^. This work used an ex vivo spinal cord preparation from young animals, reconstructing morphology from patch clamp recordings, limiting the sample to superficial PNs (lamina I) identified by recovery of contralateral projecting axons. The same group have also reported on putative projection neurons outside LI, though still limited to populations close to the spinal cord surface to enable visualised patch clamp recording in the ex vivo spinal cord. This sample included cells in the Lateral Spinal Nucleus, with a subpopulation (*n* = 4) exhibiting bilateral axonal projections and asymmetrical dendritic trees preferentially oriented to the lateral and ventral planes^65^. Similarly, axons entering the dorsal funiculus were also noted, with authors suggesting a potential propriospinal function. While these anatomical characteristics resemble LV^Lat^ SPBNs, the LV^Lat^ SPBNs reside deep within the lateral white matter making them inaccessible and unlikely to be included in biocytin reconstructions from the ex vivo spinal cord preparation. Nevertheless, this similarity suggests projection neurons located outside the lateral spinal grey matter boundaries share similar properties, raising the possibility of a common developmental origin. Regarding PNs in the deep DH, to our knowledge the only description of local axon collaterals arising from these cells comes from reconstructions a sample of virally labelled mouse SPBNs following patch clamp recordings in spinal cord slices, though these cells were located in the medial portion of the deep DH, outside the highly myelinated region containing LV^Lat^ SPBNs^12^. The current experiments showing local collateral branching and LV^lat^ SPBN photostimulation elicited activation in DH circuits, including input into the superficial DH, provides the first functional confirmation that collateral branches arising from these cells have the potential to influence a range of spinal processing functions.

Importantly, there are broad implications for the diverse LV^Lat^ SPBN collateral signalling territories identified by our experiments. Prominent ipsilateral activation of SDH and DDH circuits by LV^lat^ SPBN photostimulation is consistent with the above neuroanatomical evidence of local axon collaterals from LI projection neurons, whereas bilateral activation was more surprising. Despite this, work has established that LI projection neuron axon collaterals are very complex in their trajectory and can occupy intersegmental, intrasegmental, and contralateral spinal tissue^13,14^. Another unexpected region to exhibit activation following LV^Lat^ SPBN recruitment was LX, an area often associated with ascending visceral nociceptive signals^66^. While the trajectory of projection neuron axons is well known to pass this region as they cross to the contralateral spinal cord before ascending in the anterolateral tract, signalling from projection neurons in this area has not been described. Thus, in addition to relaying spinal output signals to the brain, activation of the LV^lat^ SPBN population has the capacity to elicit activity in several spinal circuits implicated in a range of functions. The fact that signals relayed from LV^lat^ SPBNs to the brain are also shared with these circuits suggests an important role in coordination. In support of this model, recent work argued that LI projection neurons may directly modulate motor reflex activity via ventrally projecting axon collaterals, as well as contralateral projecting collaterals terminating in premotor territories^13^.

The functional outcome of signalling from local LV^lat^ SPBN axon collaterals critically depends on the identity of cells receiving this input and although our data does not directly address this issue, the electrophysiology of recorded neurons does suggest many cells receiving monosynaptic LV^lat^ SPBN input exhibit an inhibitory interneuron phenotype^39–41,43^. Consistent with this interpretation our data also resolved some photostimulation evoked inhibitory inputs with polysynaptic characteristics. These inputs were rare but must be considered against our use of a transverse spinal slice preparation where many axons from LV^Lat^ SPBNs would be truncated, likely requiring direct terminal photostimulation to evoke synaptic release and postsynaptic responses. Furthermore, these inputs must then evoke AP discharge in postsynaptic cells to produce polysynaptic responses. Thus, we view the prevalence of polysynaptic inhibitory input in this study an underestimate of these connections.

Targeting of inhibitory interneurons is a common motif for axon collaterals arising from output/projection neurons in many CNS regions. This includes the motoneuron and Renshaw cell relationship in the ventral spinal cord^24^, as well as Principal cells of the Piriform cortex which receive feedforward and feedback inhibition from neighbouring inhibitory interneurons relevant to olfactory coding^22^. In the DH, inhibition is critical for segregating tactile and nociceptive circuits^38,67,68^, with several interneuron populations implicated in presynaptic inhibition of primary afferents^44,68^ as well as post-synaptic inhibition of excitatory circuits^68,69^. Thus, LV^lat^ SPBN collaterals may engage these circuits implicating them in spinal gating^3^. In line with this, recent work has shown that deep DH SPBNs are targeted by vGLUT1 positive primary afferents^52^, providing a substrate for low-threshold input to activate these cells, and in turn recruit inhibition within nociceptive circuits. Alternatively, LV^lat^ SPBN collaterals may engage feedback inhibitory circuits to regulate their own excitability as occurs with recurrent inhibition between Renshaw cells and motor neurons^23^. Finally, inhibition engaged by these collaterals could be important for terminating ascending sensory signals destined for the brain as well as refining receptive fields in a centre surround fashion, well established in other sensory systems^70–72^.

In conclusion, this study confirms LV^lat^ SPBNs share information back into local spinal cord circuits. Thus, concurrent to transmitting information to supraspinal areas, collateral signalling occurs within the spinal cord, including the nociceptive circuitry of the SDH. This deep to superficial DH link appears to preferentially recruit inhibition, however, the incidence of polysynaptic circuits indicates that feedforward excitation is also possible. Together, this data highlights an underappreciated aspect of PN function in spinal sensory processing with widespread local and intersegmental actions. As the function of local SPBN collaterals continues to be revealed, this efference copy will likely become an important addition to spinal sensory processing models. Future studies will be required to better understand the complex and diverse nature of these collateral circuits, help clarify the behavioural roles they play, and determine implications for chronic pain and other sensory pathologies.

### Methods

All surgical and experimental procedures were approved and undertaken in accordance with the University of Newcastle animal care and ethics committee and adhered with the ARRIVE guidelines^73^. These experiments used wild-type C57BL/6 mice (4–8 weeks old both sexes), housed in an animal care facility with continuous access to food and water *ad libitum* under a 12-h light/dark cycle. For neuroanatomical experiments mice (*n* = 9) received unilateral parabrachial nucleus injection of the retrograde enhanced adeno-associated virus (rAAV2-retro-hsyn-ChR2 ~ eYFP, or rAAV2-retro-hsyn-eYFP - herein termed rAAV2-ChR2 and rAAV2-GFP respectively) in combination with AAV9-Cb7-Cl-mCherry (AAV9-RFP), injection of AAV9-RFP alone (*n* = 3), or the tandem injection of retro-AAV-Cre and brainbow to the PBN and spinal cord (*n* = 4), respectively. Sources, titres and specific virus details can be found in Table S1. For electrophysiological experiments, animals (*n* = 16) received bilateral PBN injections of rAAV2-retro-hsyn-ChR2 ~ eYFP in the to maximise expression of ChR2 in spinoparabrachial projection neurons (SPBNs) for subsequent spinal slice electrophysiology experiements.

### Intracranial and intraspinal viral injections

Mice underwent surgery for intracranial injection to transduce fluorophore labels (RFP, GFP), optogenetic probes (ChR2), or Cre recombinase (Cre). Briefly, mice were anaesthetised (isoflurane: 5% induction, 1.5–2% maintenance) and secured in a stereotaxic frame (Harvard Apparatus, Massachusetts, U.S.A). Crainiotomies provided access for unilateral (anatomical experiments) or bilateral (electrophysiology experiments) PBN injection of ~ 700 nL of viral sample over 5 min via Picospritzer (PV820, WPI, Florida, USA) or nanoject (Harvard Apparatus, USA) expulsion. For Cre injections, smaller volumes were used (300nL). Stereotaxic coordinates for craniotomies were 5.25 mm posterior to bregma, 1.2 mm lateral to the midline, and at a depth of 3.8 mm from the skull surface, adapted from The Mouse Brain Atlas^74^; (Fig. 1). The pipette was left in place for 7–10 min after injection to minimise drawing the virus sample along the pipette track. Based on other studies where viral labelling of cells with long axons has been reported^75^ and our own observations^12^ mice were maintained for a 2-week post-injection time to allow sufficient retrograde viral transduction in SPBNs before anatomical or electrophysiological analysis. All animals made uneventful recoveries and showed no overt behaviours to indicate poor surgical outcome or pathology.

Mice that had received cranial injections of Cre also received a spinal injection of Cre-dependent AAV-Brainbow in the same surgery. The method for spinal injection was adapted from Haenraets et al.^76^. Briefly, a single injection targeted the intervertebral window of T13 which overlies the 4th lumbar segment. The vertebral column was held with a Cunningham spinal adaptor (Harvard Apparatus: #51690) attached to the stereotaxic frame. The spinal cord was exposed, with the midline demarcated by the dorsal central blood vessel, and 500 nL of AAV9-Brainbow was injected in the lumbar region 300 μm lateral to the midline at a depth of 200 μm from the spinal cord surface using a glass injection pipette attached to a Nanoject iii (Drummond Scientific Company, Broomall, PA, USA). The pipette was maintained in place for ~ 5 min to minimize the chance of virus spread along the pipette track before it was retracted. Animals were recovered from anaesthesia before being transferred to their home cage with a heat map to support post-operative recovery and analgesia (0.1 mg.kg buprenorphine administered subcutaneously).

In anatomical experiments, the brains of all virally injected animals were sectioned to verify the inclusion of the lateral parabrachial nucleus within the injection site, following perfusion fixation (Fig. 1). Brains were cryostat sectioned (50 μm, Leica CM1900), with sections washed in 0.1 M PBS, before undergoing a blocking step in 0.3 M PBS with 10% Normal donkey serum (v/v), and 0.3% triton X. Brain sections across the span of the PBN were taken and virally transduced signal was boosted using antibodies against GFP and RFP incubated overnight in 0.3 M PBS with 10% NDS and 0.3%Triton-X. Following 3 × 30 min 0.1 M PBS washes, brain sections were transferred to host specific secondaries in 0.3 M PBS with 0.3%triton-x and incubated overnight. For comparison of neuron transduction by AAV9 and rAAV2 serotypes across the injection site, a representative PBN slice was selected and imaged at 10x on an Olympus BX51 with CCD camera, exposure time maintained constant across animals. Neurons labelled by each serotype (AAV9 = RFP, rAAV2 = GFP) were counted and compared in these images. To compare process labelling (axon/dendrite) and fluorescence achieved be each serotype at the injection site, the same 10x image was overlaid with a bright field image to demarcate the boundaries of the lPBN and the SCP, before taking a mean pixel/grayscale intensity in the same areas of both channels.

### Quantification and anatomical analysis of lateral LV SPBNs

The relative incidence of SPBNs in the lateral LV region was assessed in sagittal sections taken from the spinal cords of AAV9-RFP PBN injected animals, reacted with Anti-RFP and Anti-NeuN. At least 2 sections (50 μm) were taken from the lateral dorsal horn where the dorsal band of grey matter was narrow and the longitudinal reticulated fasciculi were obvious. In some instances it was necessary to exclude sections of images that were transitional, therefore the numbers presented are not absolute. Imaging was undertaken on a Crest Optics x-light V3 spinning disk confocal at 20x magnification (field size: 675 μm x 675 μm). Display of the axonal territories was the result of a maximal intensity projection (28 μm, z = 1 μm) of an rAAV2-GFP spinal section, taken at 20x on a Zeiss LSM900 confocal with Airyscan 2. The morphological characteristics of LV lateral SPBNs were also studied in animals that received an intersectional labelling strategy of rAAV2-Cre-GFP injection in PBN and intraspinal injection of AAV-Brainbow. Sagittal sections were taken, reacted for Anti-MCherry, Anti-BFP and Anti-TFP to reveal Brainbow labelling, and imaged on a Zeiss LSM900 with Airyscan2 at 20 × (0.8x zoom) or 40x objective. Importantly, in the absence of viral Cre, brainbow labeling was absent following spinal injection. Three colour brainbow transduction provided unique colour labelling of individual neurons to allow characterisation of somatodendritic morphology based on soma size, and dendritic complexity. Unique colour profiles were traced using FIJI’s Simple Neurite Tracing plugin^77^ to assess morphological parameters including soma area, number of primary dendrites, dendritic length, and dendritic branching. Dendritic territories were also analysed, with rostrocaudal extent (RC) defined as the distance (in µm) between the rostral and caudal-most dendritic terminations. Ventral extent was the distance from the centroid of the cell soma to the ventral-most dendritic termination, and Dorsal extent was the distance from the cell soma centroid and the dorsal-most centroid. Dorsoventral extent (DV) was the sum of ventral and dorsal extents. The relative distribution of dendrites compared as a ratios for the rostrocaudal and dorsoventral planes (RC/DV), and dorsoventral plane (D/V). The mediolateral dendritic territory was assessed across multiple image stacks due to sagittal section orientation, with four sections consistently capturing dendritic profiles. For this plane, the total dendritic length in the section containing the soma, the section medial, and two sections lateral to this region were compared.

### In vivo photostimulation and phosphorylated ERK analysis

For analysis of spinal photostimulation outcomes using phosphorylated ERK immunolabelling, mice that had previously received unilateral viral injection (including rAAV2-ChR2) in PBN (*n* = 9) were anaesthetised (Isoflurane: 3% induction, 1.5-2% maintenance) and positioned on a feedback temperature controller (37 °C). The spinal cord was exposed with a dorsal approach, removing connective tissue and paraspinal musculature overlying thoracic vertebra 12 (T12). One side of the T12 vertebra was thinned, and a partial laminectomy exposed the dorsal lumbosacral enlargement contralateral to the PBN injection site, leaving the bone over the ipsilateral spinal cord in place. To account for the deep and lateral location of AAV2-ChR2 transduced SPBNs, the laminectomy was extended laterally to maximise photostimulation exposure of these cells. A terminal anaesthesia syringe (containing sodium pentobarbitone) was inserted in preparation for overdose at the conclusion of the photostimulation protocol and anaesthesia was maintained for a further 30 min to minimise any ERK phosphorylation associated with surgery. An optic fibre (Thor labs, 0.4 mm, 470 nm, 16mW) was positioned over the exposed lateral spinal cord, and continuous photostimulation was applied for 4 min (10ms pulses, 10 Hz stimulus; 10% duty cycle). Following an additional 5-minute delay, allowing development of photostimulation-related ERK-phosphorylation, a 50 mg/kg sodium pentobarbitone overdose was administered (i.p.). Animals were then rapidly transcardially perfused with 0.9% saline, followed by 4% paraformaldehyde (PFA) in 0.1 M phosphate buffer (PB). Lumbosacral spinal cords and brains were isolated and postfixed for a further 2 h in 4% PFA in 0.1 M PB, washed (3 × 15 min) and stored in 0.1 M phosphate buffered saline. Spinal cords were sectioned (50 μm, Leica CM1900) in the transverse plane and subsequently reacted with a primary/secondary antibody combinations to boost endogenous ChR2-GFP and immunolabel pERK. Primary antibodies were diluted in 0.3 M PBS with 0.3%Triton-X. Primary and secondary antibody reactions proceeded overnight, with three 30-minute PBS washes following each reaction. Sections were mounted in Flurogel media (with DABCO fluorescence stabiliser; Proscitech, Thuringowa, QLD, Australia: IM037) and imaged using a Zeiss LSM900 with airyscan (10x or 20x air objective, z = 1 μm, Pinhole: 1AU, zoom = 0.8). Neurons were visualised and counted from images using the ImageJ cell counter plugin taskbar to assign x/y coordinates to each SPBN and/or pERK positive cell profile^78^. Neurons were only included in the analysis if they appeared in 4 consecutive optical sections.

### Electrophysiology and ChR2-assisted circuit mapping

Spinal cord slices were prepared for patch-clamp electrophysiology and ChR2-assisted circuit mapping (CRACM) using previously reported techniques^39,43,79,80^. Briefly, animals were anaesthetized with ketamine (100 mg/kg *i.p*.) and decapitated. The ventral surface of the vertebral column was exposed, and the spinal cord rapidly removed (within 8–10 min) in ice-cold sucrose substituted cerebrospinal fluid (ACSF) containing (in mM): 250 sucrose, 25 NaHCO_3_, 10 glucose, 2.5 KCl, 1 NaH_2_PO_4_, 1 MgCl_2_ and 2.5 CaCl_2_, bubbled with carbanox (95% O_2_, 5% CO_2_) to achieve a final pH of 7.3–7.4. Transverse spinal cord slices (LI - L5 segments, 300 μm thick) were prepared using a vibrating microtome (Campden Instruments 7000 smz, Loughborough, UK), transferred to an interface incubation chamber containing oxygenated ACSF (sACSF with 118 mM NaCl substituted for sucrose), and allowed to equilibrate at room temperature for at least one hour prior to recording. The brain of each animal was also removed sectioned, and inspected under fluorescent illumination to confirm that the PBN was included in the injection site. Recordings were only collected in animals where the PBN contained viral expression.

Spinal cord slices were transferred to a recording chamber maintained at room temperature (22–24 °C) and continuously superfused with ACSF. Retrogradely-labelled SPBNs were identified by ChR2/eYFP expression under fluorescence illumination and targeted for recording using near-IR differential interference contrast optics. Recordings were also acquired from unidentified neurons throughout the spinal DH to test for photostimulation evoked synaptic input arising from ChR2-expressing SPBNs. Patch pipettes (4–8 MΩ; Harvard glass) were filled with a potassium gluconate based internal solution containing (in mM): 135 C_6_H_11_KO_7_, 8 NaCl, 10 HEPES, 0.1 EGTA, 2 Mg_2_ATP, 0.3 Na_3_GTP, (pH 7.3 with KOH, and liquid junction potential = 14.7 mV). All data were collected using a Multiclamp 700B amplifier (Molecular Devices, Sunnyvale, CA, USA), digitized online (sampled at 10 kHz, filtered at 5 kHz) using an ITC-18 computer interface (Instrutech, Long Island, NY, USA), acquired and analysed using Axograph X software (Molecular Devices, Sunnyvale, CA, USA). Input and series resistance (< 40 MΩ) was monitored throughout all recordings and data were excluded if these values changed by more than 20%. No correction was made for the calculated liquid junction potentials.

Photostimulation was produced by a high intensity LED light source (CoolLED PE-2, Andover, UK) delivered through the microscope optics and controlled by Axograph X software^38,79^. Recordings from ChR2 positive SPBNs assessed the ability of virally transduced cells to reliably evoke action potential discharge in the on-cell recording configuration to reflect the SPBN status in subsequent CRACM experiments (i.e. without the electrical load of the recording pipette and infusion of pipette solution). Recordings from unidentified neurons were assessed for optically evoked excitatory postsynaptic currents (oEPSCs) arising from ChR2-YFP-expressing SPBNs in voltage clamp (holding − 70mV) using a protocol that delivered either 1ms–1s photostimulus at 0.083 Hz (both 16mW). The 1s stimulus was applied as a long search stimulus, and if a photostimulus derived input was identified, all characterisation was undertaken with a 1ms stimulus. The potential for polysynaptic optically evoked inhibitory postsynaptic currents (oIPSCs), recruited by ChR2-expressing SPBNs, was assessed using the same protocols with holding potential adjusted to -40mV to unmask inhibitory (outward) currents. In most cases 4-aminopyridine (4-AP) was bath applied (200µM) to enhance terminal neurotransmitter release from photostimulated axons^81^. Cumulative peristimulus histograms of EPSC/IPSC frequency were first constructed for 10 consecutive photostimulation trials (bin width = 50ms), with mean frequency (µ) and standard deviation (σ) calculated outside the photostimulation period in each recording. These values were used to set a criterion threshold (µ + 4σ) that EPSC/IPSC frequency was required to exceed following photostimulation, to be considered receiving a SPBN collateral-evoked input. In records deemed to contain a photostimulation-evoked response, oEPSC/oIPSC latency from photostimulation onset, and amplitude were calculated for each trial. A threshold of 8ms was then applied to the average latency of all inputs to differentiate putative monosynaptic and polysynaptic optically evoked postsynaptic currents (monosynaptic oPSC latency < 8ms; polysynaptic > 8ms). This threshold was derived from mean value (+ 4SD) of the recruitment delay between photostimulation onset and AP initiation in ChR2-expressing SPBNs (2.88 ± 0.67 ms, Fig. 7D), with 2 ms added for conduction and synaptic delays taken from previous paired recording studies in the dorsal horn^82^. In some instances, oEPSC sensitivity to bath applied AMPA/kainite receptor antagonist 6-cyano-7-nitroquinoxaline-2,3-dione (CNQX) was assessed.

A range of electrophysiological properties were also recorded from the unidentified DH sample to characterise these neurons. Membrane capacitance and input resistance were calculated from the averaged current response (30 trials) during a 5mV hyperpolarising step in voltage clamp (holding potential − 70 mV). AP discharge was evaluated in current clamp mode by injecting depolarising current steps (20 pA increments, 1 s duration) from a membrane potential of -60 mV^12,39^. Characteristics of AP discharge were measured from the response two steps above rheobase (i.e., + 40pA above the first step to evoked AP). All APs were detected using a derivative threshold (dv/dt = 15mV/ms) and the voltage at this detection point was defined as AP threshold. AP number was the sum of all APs detected in an individual step response, spike latency was the time from step onset to the first AP, discharge duration was the difference between latencies of the last and first AP in a step response, instantaneous frequency was defined as the reciprocal of the inter-event interval between successive APs, adaptation was the ratio of the first and last AP instantaneous frequency, and attenuation was the ratio of the first and last AP peak. The overall pattern of AP discharge was classified based on existing schema for lamina II neurons^36,39,42^. Delayed firing (DF) responses exhibited a clear, ramped voltage response at onset and delay before AP discharge. Tonic firing (TF) was characterised by continuous AP discharge for the current step duration. Initial bursting (IB) responses exhibited an AP burst at current onset and sometimes a rapid onset depolarising hump at rheobase. Single spiking (SS) responses exhibited a single AP at current step onset, regardless of step amplitude. Gap firing (GF) exhibited an AP at onset, followed by a ramped voltage response before further AP discharge. Phasic (P) discharge was characterised by periods of AP discharge interrupted by breaks. Reluctant firing (RF) was assigned to neurons that did not discharge APs despite sustained depolarisation above threshold (~ -20 mV) but did exhibit APs when the step protocol was repeated from a depolarised membrane potential (approximately − 50 mV).

Spontaneous excitatory postsynaptic currents (sEPSCs) were recorded in voltage clamp mode (holding potential of -70mV) for a minimum of 60-seconds. sEPSCs were detected using a sliding template method (a semi-automated procedure in Axograph X software). All detected events were inspected individually and excluded from analysis if they contained overlapping EPSCs or did not exhibit a rapid rise and slow decay phase, characteristic of synaptic currents. Average sEPSC frequency was determined over 30-seconds of recordings, dividing the number of events captured in this epoch by 30. Peak sEPSC amplitude, rise time (10–90% of peak), and decay time constant (Tau; 10–90% of the decay phase) were obtained from an averaged sEPSC of all captured events. sEPSC charge was calculated as the area under the curve of the averaged sEPSC, and excitatory synaptic drive was calculated by multiplying sEPSC charge with sEPSC frequency.

Subthreshold voltage activated currents were assessed using a voltage-clamp protocol that applied an initial hyperpolarisation step to − 100 mV (1 s duration), followed by a depolarizing step to − 40 mV (200 ms duration) with P/N leak current subtraction (Axograph X software, automated procedure). This protocol identified four voltage-activated currents previously described in DH neurons including: fast and slow forms of A-type transient outward potassium currents (I_A_)^36,83^; low threshold transient inward currents characteristic of the T-Type calcium current (Ca_T_)^43,84^; and a non-specific inward cationic current (I_h_)^42,85^.

### Statistical analysis

All data are presented as mean ± standard deviation (SD) unless otherwise stated. One-way ANOVAs were used to compare cells with monosynaptic inputs to cells with polysynaptic inputs, and unconnected cells. Shapiro Wilks test of normality and Levenes test for homogeneity of variances was assessed across samples. In cases where the assumption of normal distributions were not met, a Welchs ANOVA was used to test means. Statistical tests were applied using SPSS v26 (IBM Corp. Armonk, NY, USA) or Igor Pro 9 (WaveMetrics, Lake Oswego, OR, USA).

## Electronic supplementary material

Below is the link to the electronic supplementary material.

Supplementary Material 1

## Data Availability

The datasets generated and analysed during the current study are available from the corresponding authors on reasonable request.
