# Supplementary material for: Lateral lamina V projection neuron axon collaterals connect sensory processing across the dorsal horn of the mouse spinal cord
Source: Sci Rep. 2024 Nov 1;14:26354. doi: 10.1038/s41598-024-73620-4 (PMC11530558; doi:10.1038/s41598-024-73620-4)
Supplement: Supplementary file 1 — Supplementary Material 1 [file 41598_2024_73620_MOESM1_ESM.docx]

**Lateral lamina V projection neuron axon collaterals connect sensory processing across the dorsal horn of the mouse spinal cord**

Tyler J Browne^1*^, Kelly M Smith^2^, Mark A Gradwell^3^, Christopher V Dayas^1^, Robert J Callister^1^, David I Hughes^4^, & Brett A Graham^1^

^1^ School of Biomedical Sciences & Pharmacy, Faculty of Health, University of Newcastle, Callaghan; and Hunter Medical Research Institute (HMRI), New Lambton Heights, NSW, Australia.

^2^ Department of Neurobiology and the Pittsburgh Center for Pain Research, University of Pittsburgh, Pittsburgh, PA 15213, USA.

^3^ Department of Cell Biology and Neuroscience, Rutgers, The State University of New Jersey, Piscataway, NJ, USA; and W.M. Keck Center for Collaborative Neuroscience, Rutgers, The State University of New Jersey, Piscataway, NJ, USA.

^4^ Institute of Neuroscience Psychology, College of Medical, Veterinary & Life Sciences, University of Glasgow, Glasgow, UK.

**Text pages:**

**Figures:** 1

**Tables: 1**

**Corresponding author:**

T. J. Browne: School of Biomedical Sciences and Pharmacy, Faculty of Health, University of Newcastle, Callaghan, NSW 2308, Australia. Email: [Tyler.Browne@newcastle.edu.au](mailto:Tyler.Browne@newcastle.edu.au)

**Acknowledgements:**

This work was funded by the National Health and Medical Research Council (NHMRC) of Australia (grants 631000, 1043933, 1144638, and 1184974 to B.A.G and R.J.C), the Hunter Medical Research Institute (grant to B.A.G. and R.J.C.), and the BBSRC (grant BB/J000620/1 to D.I.H.). All authors have no conflict of interest to declare.

| Virus Class | Construct | Titre (GC/mL) | Source | Cat# | Acknowledgement |
| --- | --- | --- | --- | --- | --- |
| Opsin Probe (ChR2) | pAAV-hSyn-hChR2(H134R)-EYFP  (rAAV2-ChR2) | 1.98x10^12^ | Addgene | 26973-AAVrg | Gifted by Karl Deisseroth |
| Fluorescent Reporter | pENN.AAV.CB7.CI.mCherry.WPRE.RBG  (AAV9-RFP) | 2.5x10^13^ | Addgene | 105544-AAV9 | Gift by James M. Wilson |
| Fluorescent Reporter | pAAV-hSyn-EGFP  (rAAV2-GFP) | 2.3x10^13^ | Addgene | 50465-AAVrg | Gift by Bryan Roth |
| Cre recombinase | pENN-AAV-hSyn-HI-eGFP-Cre.WPRE.SV40 | 1.9 x 10^13^ | Addgene | 105540-AAVrg | Gift by James M Wilson |
| Brainbow | AAV-EF1a-BbTagBY | 5.3 x 10^11^ | Addgene | 45185-AAV9 | Gift from Dawen Cai & Joshua Sanes |
| Brainbow | AAV-EF1a-BbChT | 1.7 x 10^12^ | Addgene | 45186-AAV9 | Gift from Dawen Cai & Joshua Sanes |
| Antibody | **Details** | **Conc.** | **Source** | **Cat#** |  |
| Primary | Chicken antiGFP | 1:1000 | Abcam | Ab13790 |  |
| Primary | Goat antiRFP | 1:1000 | Jomar Life research | 200-101-379 |  |
| Primary | Rabbit Anti-phosphoERK | 1:1000 | Cell Signalling Technologies | #9101 |  |
| Primary | Chicken anti-mCherry | 1:5000 | Abcam | ab205402 |  |
| Primary | Rat anti-mTFP | 1:1000 | Kerafast Inc., Boston, MA USA | EMU103 |  |
| Primary | Guinea Pig anti-TagRFP | 1:500 | Kerafast Inc., Boston, MA, USA | EMU107 |  |
| Primary | Rabbit anti-NeuN | 1:500 | Abcam | 104225 |  |

Supplemental Table 1 : Resources and reagents

***
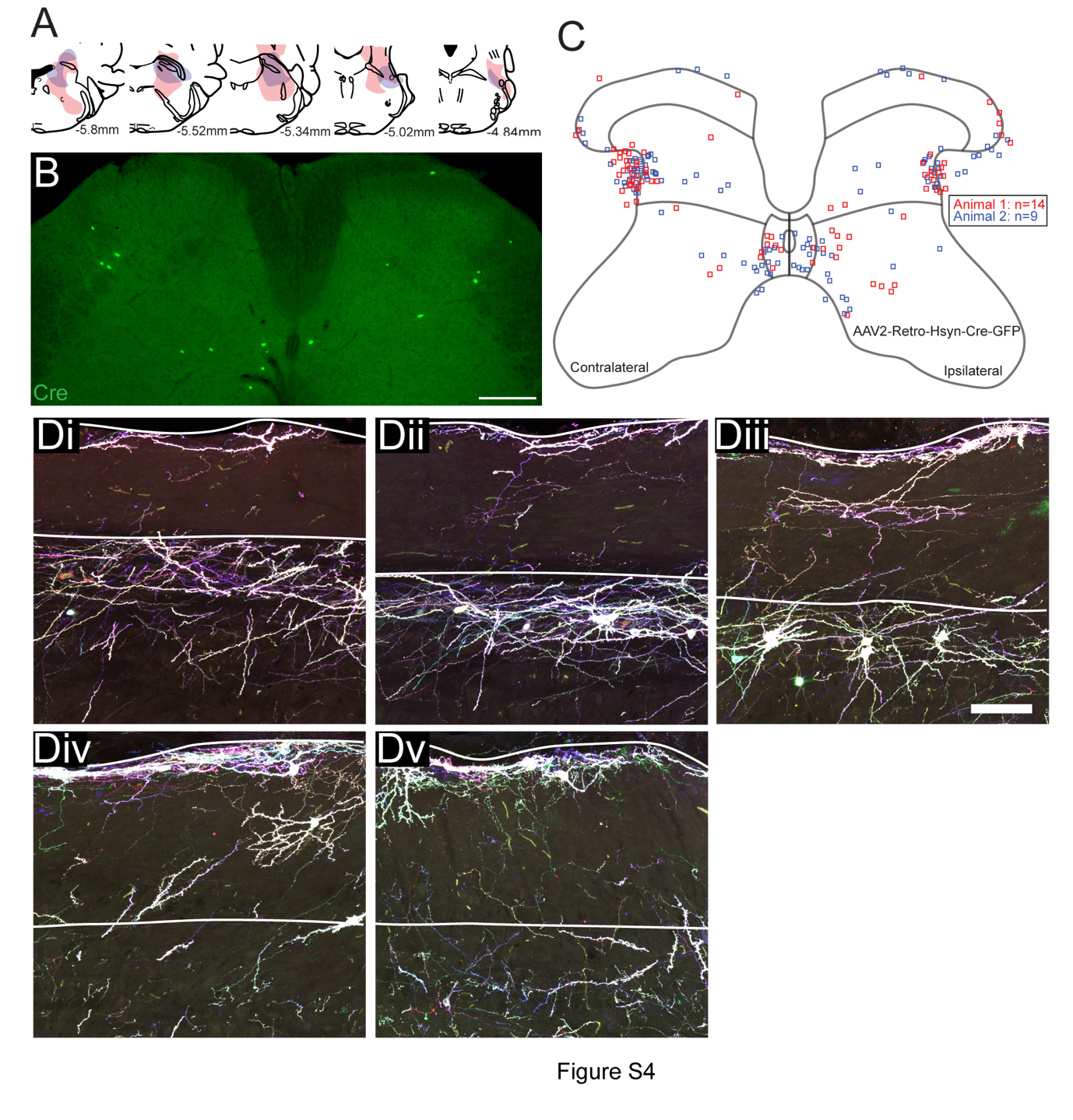
***

***Supplementary Figure 1: High efficiency of Cre leads to reduced LVLat specificity of AAV-retro constructs . A,*** maps show brain injection site reconstructions from two animals (red and blue) that received rAAV2-CreGFP injections in PBN as a trial to confirm this constructs can transduce LV^Lat^ SPBNs for subsequent Cre-dependent brainbow labelling experiments. Note, both injections spanned the PBNs rostrocaudal extent. **B,** image shows a transverse spinal cord section (50mm thick) with representative rAAV2-mediated Cre-GFP expression. Note bilateral Cre-GFP expression including profiles located in the LV^Lat^ region. **C,** summary map showing distribution of Cre-GFP labelled profiles counted across two animals (14 and 9 sections, respectively - red and blue). Labelled profiles were concentrated bilaterally in the LV^Lat^ region, as well as more sparse labelling in the superficial DH, deep DH, LX and intermediate zone. ***Di-v.*** shows a series of sagittal sections following PBN-Cre and Spinal Cord-Brainbow injection from lateral (***Di***) to medial (***Dv)*** and with LI and LV indicated (top and bottom, white line, respectively). Note the increased expression across LV^lat^ when compared to the trials (***Di-Diii***). There was increased CreGFP expression, and subsequent Brainbow expression within SPBNs in Lamina I (***Di-v***), Lamina III (***Diii-iv***), Lamina V medial (***Div***) and further in the medial DDH (***Dv***). Scale: B: 200μm. D: 100μm.
